# Critical iridium demands arising from global expansion of proton exchange membrane electrolysis

**DOI:** 10.1007/s44498-026-00088-y

**Published:** 2026-05-18

**Authors:** Bernhard Wortmann, Detlef Stolten, Heidi Heinrichs

**Affiliations:** 1 Institute of Climate and Energy Systems, Forschungszentrum Jülich GmbH, 52425 Jülich, Jülich, Germany; 2 Chair for Fuel Cells, Faculty of Mechanical Engineering, RWTH Aachen University, 52062 Aachen, Germany; 3 Chair for Energy Systems Analysis, Department of Mechanical Engineering, University of Siegen, 57076 Siegen, Germany

**Keywords:** Industrial ecology, Critical materials, Hydrogen economy, Platinum group metals, Energy transition, Material flow analysis

## Abstract

Proton exchange membrane electrolysis (PEMEL) is a key technology for producing green hydrogen, but its scalability is limited by the use of scarce materials, particularly iridium. Iridium oxide, the preferred anode catalyst in PEMEL, offers exceptional stability but is produced only as a by-product of platinum mining, with annual output around 7.5 tons. This study estimates future iridium demand for PEMEL under various global deployment scenarios and technological advances. Results show that meeting net zero targets will require both significant improvements in catalyst efficiency and access to roughly 30% of global iridium production annually. Supply shortages could arise as early as 2030, earlier than previously anticipated. The analysis also reveals that long-term iridium needs beyond 2040 are significantly underestimated. These findings underscore the urgent need for innovation in material efficiency and recycling, and the importance of integrating resource constraints into energy policy and technology planning to ensure a sustainable hydrogen transition.

**Supplementary Information:**

The online version contains supplementary material available at 10.1007/s44498-026-00088-y.

## Introduction

Green hydrogen is essential for achieving a greenhouse gas neutral energy system. Its primary applications include fuel for heavy transport, chemical feedstock, energy storage, and combustion fuel in heavy industries (Council & Company, 2021; Iea, 2023a, c; Taibi et al., 2020). Hydrogen produced by water electrolysis using renewable electricity is particularly promising for meeting these diverse needs as it enables low carbon hydrogen production and supports energy system flexibility (Nasser et al., 2022).

The commercially available water electrolysis technologies are Alkaline Electrolysis (AEL) and Proton Exchange Membrane Electrolysis (PEMEL) (Rocha et al., 2024). While AEL offers modest cost advantages due to the absence of precious metal catalysts (Gambou et al., 2024), PEMEL is expected to play a central role in future hydrogen production. Its higher power densities and superior load-following capabilities make it well-suited for integrating variable renewable energy sources (Smolinka et al., 2018; Kiemel et al., 2021). Moreover, PEMEL holds greater potential for innovation in catalyst efficiency, membrane durability, and scalability, unlike the more mature AEL technology (Holst et al., 2021).

Organizations such as the IEA, IRENA, and the Hydrogen Council have analyzed PEMEL scale-up, offering key projections for future hydrogen strategies. The IEA, for example, estimates a hydrogen demand of around 500 megatons by 2050 to meet net-zero targets (Iea, 2022). Despite differences in scope, all studies project rapid electrolysis expansion, with PEMEL expected to account for about 40% of capacity (Smolinka et al., 2018).

PEMEL technology depends on iridium as the anode catalyst, a critical and extremely scarce element with an annual global supply of around 7.5 tons (United States Geological Survey, 2023). No viable substitute currently exists, as iridium uniquely combines catalytic performance with corrosion resistance. This scarcity poses a potential bottleneck that could threaten decarbonization targets (Irena, 2020; Cherevko et al., 2016; Buttler & Spliethoff, 2018). To mitigate this risk, recent research has focused on reducing iridium loadings and enhancing PEMEL performance (Bernt et al., 2018; Alia et al., 2019; Möckl et al., 2022; Bernt et al., 2020), though the extent to which iridium dependence can be lowered remains uncertain.

The role of iridium in PEMEL expansion has been explored through various projections of demand, supply constraints, and technological progress. One of the earliest studies (Smolinka et al., 2018), focused on Germany and concluded that, without major reductions in catalyst loading, national iridium demand could exceed global supply. However, the analysis was based only on optimistic assumptions about technological progress and recycling infrastructure. Kiemel et al. (2021) expanded on this by integrating criticality assessments with capacity projections and also warned of potential bottlenecks if German targets are overly ambitious. Both studies are geographically limited and do not account for global demand or competing sectors. Riedmayer et al. (2023) projected iridium needs under varying loadings and recycling rates but assumed an increasing iridium supply, which is unlikely given its status as a byproduct of platinum group metal (PGM) mining (Brown et al., 2023). Minke et al. (2021) and Clapp and Zalitis (2023) modeled scenarios with declining catalyst loadings. While Minke modeled a single optimistic trajectory, Clapp offered two scenarios and estimated that only 1.5 tons/year of iridium would be available for PEMEL, based on internal knowledge of the company Johnson Matthey. Despite their insights, none of these studies fully quantify how much of the iridium supply can be allocated to PEMEL given global market competition. Furthermore, the constructed models lack recursive calculation of iridium demands due to end-of-life replacements, leading to underestimations in demand projections.

This study addresses key research gaps by developing a recursive model that estimates iridium demand from both initial PEMEL installations and end-of-life replacements, incorporating recycling effects. It also introduces refined supply scenarios by accounting for competing market demands and iridium price trends. Demand is assessed under two capacity expansion scenarios: one based on current real-world PEMEL projects (Iea, 2024), and the other aligned with the IEA’s Net-Zero Emissions pathway (Iea, 2023c).

## Method

**Fig. 1:**
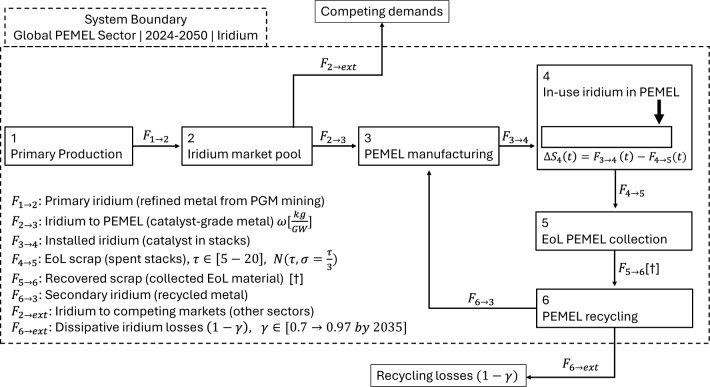
Dynamic material flow model for iridium in the global PEMEL sector (2024–2050). Numbered boxes represent system processes; labeled arrows represent iridium mass flows between processes, indexed by origin and destination process number. The stock change in process 4 (in-use iridium) is defined as $\Delta S_4(t) = F_{3\rightarrow 4}(t) - F_{4\rightarrow 5}(t)$. Flows exiting the system boundary include iridium allocated to competing markets ($F_{2\rightarrow {\text {ext}}}$, exiting at process 2) and dissipative recycling losses ($F_{6\rightarrow \textrm{ext}}$, exiting at process 6), given by $(1-\gamma )\cdot F_{5\rightarrow 6}$. Primary iridium supply is assumed constant at $7.5~\mathrm {t~yr^{-1}}$. † The recovery parameter γ is applied as a single aggregated factor spanning both EoL collection (P5) and recycling processing (P6), i.e. $\gamma = \gamma _\textrm{col} \cdot \gamma _\textrm{rec}$, and the model does not resolve these two efficiencies separately (see Equation 4). γ is assumed to increase linearly from 0.70 to 0.97 by 2035. Consequently, the scrap stream $F_{5\rightarrow 6}$ represents an aggregated EoL material flow rather than an explicitly tracked scrap commodity; manufacturing scrap and iridium losses during MEA and stack fabrication are not resolved as separate flows and are assumed negligible relative to the EoL scrap volumes over the modeled horizon

This section outlines the method used to assess iridium demand, potential supply bottlenecks, and key sensitivities. It presents the core equations and model parameters varied across different scenarios, embedded in a dynamic material flow model that explicitly captures stock-flow interactions, end-of-life recycling, and competing sectoral demands for iridium (Figure 1), with exact process definitions given in Supplementary Information SI 1 (Sect. S2, Table S1). A detailed illustration of the underlying algorithm is provided in the Supplementary Information SI 1 (Sect. S1, Figure S1). A direct comparison between the dynamic (recursive) and non-dynamic (non-recursive) formulations is presented in the Supplementary Information SI 1 (Sect. S3).

### Modeling iridium demand

The total primary iridium demand in year *i* is defined as the sum of demand from capacity expansions $m_{cap}^i$ and end-of-life replacements ($m_{EOL}^i$), minus the recycled iridium available that year $m_{recycling}^i$ (Equation (1)).

1 $$\begin{aligned} m_{total}^i = m_{cap}^i + m_{EOL}^i - m_{recycling}^i \end{aligned}$$

The demand from PEMEL capacity expansion in year *i*, $m_{cap}^i$ is calculated as the product of the annual capacity additions $P_{el}^i$ and the iridium material intensity $\omega ^i$ $[kg \cdot GW^{-1}]$. The parameter $\omega ^i$ reflects the iridium required to install one unit of capacity in year *i*.

2 $$\begin{aligned} m_{cap}^i = P_{el}^i \cdot \omega ^i \end{aligned}$$

Based on this, the number of installed electrolyzers in year *i* is calculated by dividing the annual capacity target by the average electrolyzer size of 1 MW (Reksten et al., 2022). Each unit is assigned a lifetime drawn from normal distribution with mean τ, a specified standard deviation $\sigma = 1/3 \tau$, and bounded lifetime limits $[1,2\cdot \tau ]$. This reflects real life variability in operational conditions and manufacturing quality. Based on these lifetimes, end-of-life years are estimated to calculate both replacement demands $m_{EOL}^i$ and recycled iridium $m_{recycling}^i$. The required replacement capacity in year *i* is derived from the capacity installed τ years earlier, denoted as $P^{i-\tau }_{el}$. The resulting replacements demand $m_{EOL}^i$ is given by Equation 3. Newly installed units also receive sampled lifetimes, continuing the process recursively through the full analysis time horizon.

3 $$\begin{aligned} m_{EOL}^i = P_{el}^{i-\tau } \cdot \omega ^i \end{aligned}$$

The recovered iridium quantity is calculated by multiplying the end-of-life scrap volume by a recycling efficiency factor $\gamma ^i \in (0,1)$ which simultaneously reflects both the technical efficiency of the recycling process and the fraction of catalyst material that is successfully collected after use. This assumes negligible iridium loss during operational life. The resulting expression is given in Equation (4).

4 $$\begin{aligned} m_{recycling}^i = (m_{cap}^{i-\tau } + m_{EOL}^{i-\tau }) \cdot \gamma \end{aligned}$$

In summary, iridium demand is driven by four key parameters: iridium material intensity $\omega ^i$, electrolyzer lifetime τ, capacity expansion $P^i_{el}$, and recycling rate $\gamma ^i$. Their parametrization is detailed in the following subsections.

### Global capacity expansion scenarios

**Fig. 2:**
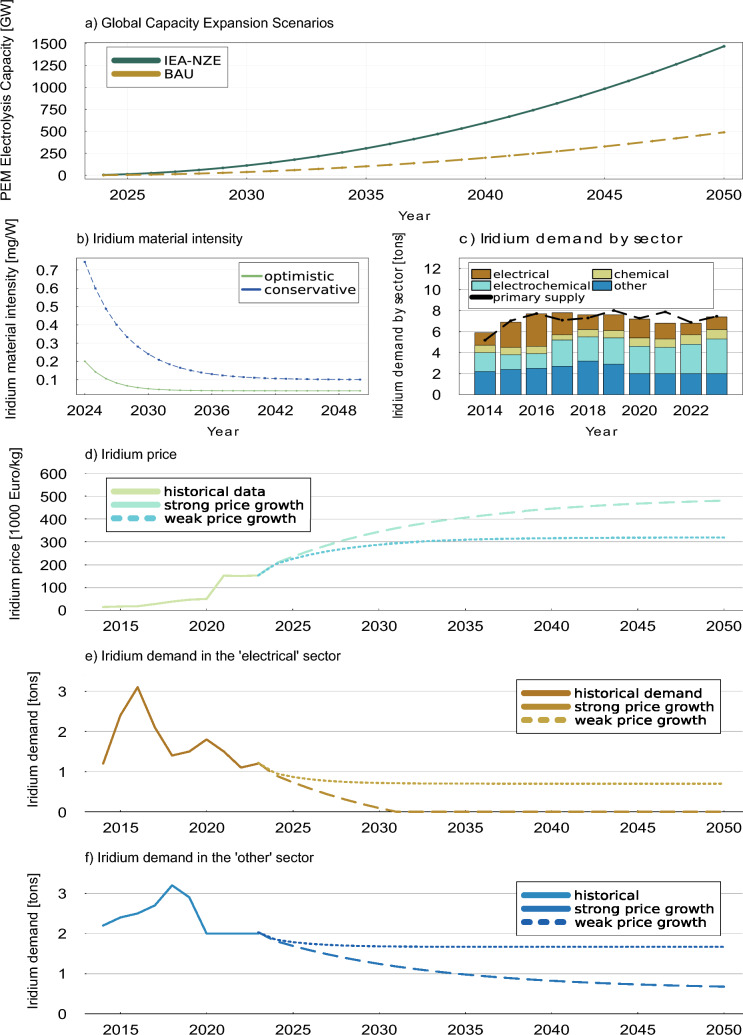
**a)** Capacity expansion for PEMEL up to 2050 (Iea, 2024, 2023c). **b)** Evolution of iridium material intensity as in Clapp and Zalitis (2023). **c)** Supply and demand of iridium by sector from 2014 to 2023 (Brown et al., 2023) and supply (United States Geological Survey, 2023). **d)** Iridium price forecast. **e)** Demand forecast "electrical" sector. **f)** Demand forecast "other" sector. Dampings are chosen such that a strong price growth (ϕ=0.9) and a weak price growth (ϕ=0.8) is modeled. The underlying data for each figure are available in Supplementary Information SI 2 (spreadsheet tab “Method”)

The first parameter for estimating iridium demand is the annual rate of PEMEL capacity installation. Two scenarios are used. The first is based on real and planned PEMEL projects compiled in the IEA database, which covers global developments till 2030 (Iea, 2024) and is referred to as Business-As-Usual scenario (BAU). Capacity growth is extrapolated linearly to 2050, reaching 489 GW, nearly comparable to the IEA-APS scenario target of 580 GW, which reflects national climate pledges (Iea, 2023a). The second scenario is the IEA Net Zero Emissions (IEA-NZE) pathway (Iea, 2023c), which targets net-zero emissions by 2050 and assumes a significantly larger role for PEMEL. Based on prior studies (Smolinka et al., 2018), PEMEL is projected to capture 40% of the total electrolyzer market, resulting in 1468 GW by 2050 (see Figure 2a).

The two scenarios differ substantially in their global 2050 capacity targets. The BAU reflects a continuation of current trends, while the IEA-NZE scenario represents the effort required to meet net-zero goals. Comparing them reveals how primary and secondary iridium supply must scale to enable such ambitions.

### Iridium material intensity and PEMEL lifetimes

A key parameter for estimating iridium demand is the iridium material intensity $\omega _i$ $[kg \cdot GW^{-1}]$, which reflects how efficiently iridium is utilized within a PEMEL stack. Literature values for $\omega ^i$ vary widely from 0.34 to 2.0 $mgW^{-1}$ in 2024 and there is no consensus on realistic benchmarks (Smolinka et al., 2018; Babic et al., 2017; Carmo et al., 2019; Sparber et al., 2021; Clapp & Zalitis, 2023). Target values also differ, often lacking accompanying degradation data. As Clapp et al. note, lower iridium loadings tend to reduce stack lifetime, raising concerns about long-term viability (Clapp & Zalitis, 2023). Based on degradation considerations, Clapp et al. derive a conservative lower bound of approximately 0.65 $mg/cm^2$ for a 10-year lifetime by relating dissolution rates measured by Yu et al. (2020) at ultra-low areal loadings (0.08 $mg/cm^2$) under intentionally high operating voltages of 1.9 V.. However, the relationship between the iridium material intensity and lifetime remains uncertain due to ongoing technological developments and limited operational data. This study uses the approach of Clapp et al., using exponentially decaying curves to model future iridium-specific power densities under two scenarios (Clapp & Zalitis, 2023). The *conservative* scenario assumes limited technological progress and slow reductions due to degradation constraints. The *optimistic* scenario reflects accelerated improvements from advances in catalyst design (see Figure 2b). These trajectories are used to estimate iridium demand under differing technological assumptions. Because lower iridium-specific power densities may affect system durability, model calculations also vary the average electrolyzer lifetime τ between 5 and 20 years to assess its impact on overall iridium demand.

### Recycling

Reliable estimates of iridium recycling rates remain uncertain, as noted in prior studies (Minke et al., 2021; Clapp & Zalitis, 2023). A useful benchmark has been drawn from platinum, a closely related PGM with similar recycling incentives. As highlighted by the Johnson Matthey PGM review (Brown et al., 2023), platinum recycling rates in auto catalysts range from 50-70%, despite occurring in open-loop systems, where materials are not returned to the original producer (Market News, 2023). In contrast, iridium in PEMELs is recycled in closed-loop systems (Brown et al., 2023), making a 70% recycling rate a reasonable current estimate.

How iridium recycling could scale toward its technical maximum of 95-97% (Kiemel et al., 2021; Carmo et al., 2019) remains uncertain. Given iridium’s high value and scarcity, manufacturers have strong incentives to improve recovery, particularly from end-of-life electrolyzers. Following Clapp et al., this study assumes a linear increase in recycling efficiency from 70% to 97% by 2035 to reflect likely technological progress (Clapp & Zalitis, 2023). Forecasting industrial developments of this scale involves significant uncertainties, particularly as companies often keep recycling technologies and capacities confidential leading to vast uncertainties in the estimation of accurate recycling rates. To account for these uncertainties, sensitivity calculations for different recycling rates are performed.

### Iridium supply estimates

Iridium is one of the rarest elements in Earth’s crust, with an annual global primary production of about 7.5 tons (United States Geological Survey, 2023). Its supply is highly volatile, influenced by factors such as mining strikes (Council & Africa, 2014), and is dominated by South Africa, which accounts for 90-95% of global output, followed by Russia, Canada, and Zimbabwe (United States Geological Survey, 2023). As a byproduct of other PGM mining, its future production is difficult to project. Therefore, this study assumes a constant annual supply of 7.5 tons. Since the mid 2010s, production has mostly matched demand (Brown et al., 2023), occasionally exceeding it and probably enabling limited stockpiling (see Figure 2c).

Iridium demand spans four main sectors namely electrical, electrochemical, chemical, and others. Demand in the electrical sector is largely driven by applications like spark plugs and lithium crystal growth (Brown et al., 2023). Spark plugs gained popularity in the early 2010s due to durability and low iridium prices, but recent price increases have led to growing substitution with platinum (Brown et al., 2023).

Demand in the chemical sector has remained stable and is expected to persist (Brown et al., 2023), as ruthenium-iridium catalysts play an irreplaceable role in key processes. Thus, this sector is not expected to free up iridium for PEMEL use. Iridium demand in the electrochemical sector has grown due to PEMEL deployment and copper foil production via electrolytic deposition. As copper foil is essential for lithium-ion batteries, demand for copper foil is expected to rise with battery expansion (Iea, 2023b). However, rising iridium prices may shift production toward rolling methods (Lu et al., 2024), potentially easing iridium pressure and freeing supply for PEMEL. In addition, the electrochemical sector could see further iridium demand from proton exchange membrane fuel cells (PEMFCs), where iridium-based co-catalysts are employed under specific operating conditions, such as voltage reversal mitigation (Fathi Tovini et al., 2021). While this contribution is currently secondary compared to PEM electrolysis, it highlights the broader competition for iridium across hydrogen technologies. Quantifying potential PEMFC-related iridium demand is beyond the scope of this study.

The last sector of importance which is referred to as ”other” comprises a vast number of different kinds of products where among others, some examples are jewelry and applications in dental medicine (Brown et al., 2023).

To estimate iridium availability for PEMEL, we assess price-responsive demand in the “electrical” and “other” sectors, which historically show inverse trends with iridium prices (Umicore, 2024). These two sectors are considered because they are less dependent on iridium, offer greater substitution potential, and show anticorrelated behavior with price movements, unlike the chemical and electrochemical sectors, where iridium use is often technologically essential. Price forecasts are generated using damped trend models that reduce the influence of older data over time, controlled by a damping factor ϕ (Hyndman & Athanasopoulos, 2023); see Supplementary Information SI 1 (Section S4). As shown in Figure 2d, we model two cases, strong damping (ϕ=0.8) and weak damping (ϕ=0.9), corresponding to slower and faster price growth, respectively. Values beyond ϕ=0.9 are avoided because they lead to extreme price spikes (e.g., €900,000/kg) and unrealistic demand collapse, particularly in the “other” sector. As shown in Figure 2e, demand in the “electrical” sector declines because substitutes are available. By contrast, the “other” sector, which includes diverse and often price-insensitive products such as jewelry, is harder to predict (Figure 2f). The chosen damping values therefore ensure realistic outcomes across both sectors.

## Results

### The conservative BAU scenario

**Fig. 3:**
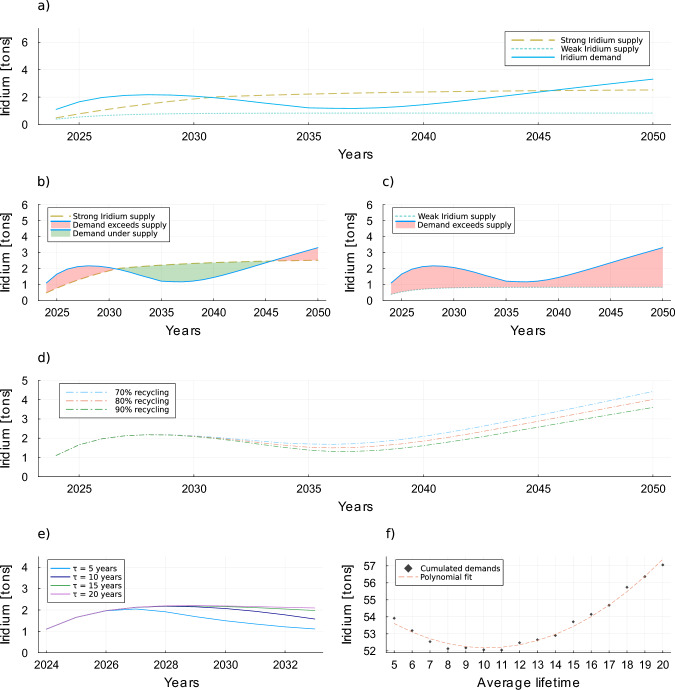
**The conservative BAU scenario**: Projected iridium demands with an average lifetime τ=10 years and recycling efficiency of 97% by 2035 and corresponding supply projections. **a)** Demand and supply projections. Values from 2024-2030 reflect demands from planned capacity expansions. **b)** Supply to demand gaps under strong supply. **c)** Supply to demand gaps under weak supply. Red areas indicate time ranges in which demand is higher than supply whereas green areas indicate time ranges where supply is higher than demand. **d)** Iridium demands from variations of recycling rates γ for 70%, 80% and 90% reached by 2035. **e)** Iridium demands within the first 9 years from variations of the average lifetime of 5, 10, 15 and 20 years. **f)** Cumulative demands for the whole time range from lifetime variations ranging from 5 to 20 years. The underlying data for each figure are available in Supplementary Information SI 2 (spreadsheet tab “BAUcon”)

In the early years of capacity expansion, iridium demand rises sharply, peaking at approximately 2.1 tons in 2028 (Figure 3a). This is followed by a decline, reaching a local minimum of around 1.1 tons in 2037, driven by improvements in iridium material intensity. Thereafter, demand steadily increases again, driven by accelerated capacity additions and growing replacement needs from end-of-life systems, eventually reaching a maximum of 3.1 tons.

Figure 3b and 3c show the gaps between projected iridium demand and supply under the two supply scenarios. Red areas indicate periods where demand exceeds supply, while green areas represent potential stock accumulation when supply exceeds demand. These areas can be integrated to quantify additional iridium which is needed (red) or potentially storable (green). Under the *conservative* BAU scenario and the strong supply projection (Figure 3b), demand exceeds supply from the start of deployment until 2031, primarily driven by planned global PEMEL projects (Iea, 2024). This suggests that some near-term projects may already face supply risks. The cumulative shortfall during this period amounts to roughly 4.5 tons, requiring an average 7.5% increase in annual iridium supply to bridge the gap. Between 2032 and 2046, however, supply exceeds demand, enabling the theoretical accumulation of approximately 10.2 tons of iridium in stock (without accounting for economic effects), sufficient to offset the 2.2-ton shortfall expected from 2047 onward. In contrast, under the weak supply projection (Figure 3c), demand exceeds supply throughout the entire time horizon. Bridging this gap would require an additional 30.2 tons of iridium, corresponding to an average annual supply increase of over 15%.

Thus, under the weak supply projection (Figure 3c), increasing primary iridium production is the only viable way to realize the *conservative* BAU scenario. However, this constraint does not apply under the strong supply projection (Figure 3b), where temporary supply shortages can be compensated through stock accumulation.

Figure 3d-f illustrates the sensitivity of iridium demand to variations in average lifetime τ and recycling rate γ. As shown in Figure 3d, varying the ramp-up speed of recycling efficiency has little impact during the first six years of capacity expansion. This behavior reflects a typical stock-flow dynamic. During the initial expansion phase, only a negligible share of installed capacity has reached end-of-life, such that the absolute amount of recyclable iridium remains small irrespective of the assumed recycling capacity ramp-up speed. However, differences grow over time, resulting in demand variations of up to 1 ton by 2050, which represent 13% of today’s primary iridium production. This underscores the long-term importance of achieving high recycling rates to meet capacity targets.

In contrast, changes in average lifetime τ have a more immediate impact. Figure 3e shows that shorter lifetimes lead to reduced primary iridium demand in the early years (2024-2033), as earlier end-of-life triggers earlier recycling. This effect is explained by the vintage structure of the model: electrolyzers installed in earlier years are associated with higher iridium material intensities, such that their earlier retirement releases comparatively larger quantities of iridium into the recycling loop. As a result, shorter lifetimes disproportionately increase recycled iridium availability in the early expansion phase. For instance, with τ=5, the initial demand gap (Figure 3b) could be nearly halved to 2.5 tons, making it more feasible to bridge using existing stockpiles.

However, this comes at a cost: shorter lifetimes increase the total number of replacements over time. Figure 3f shows cumulative demand rising with shorter τ, with a minimum occurring around 10-11 years. Thus, while shorter lifetimes reduce near-term demand, they ultimately lead to higher cumulative material requirements over the whole time horizon.

### The optimistic BAU scenario

**Fig. 4:**
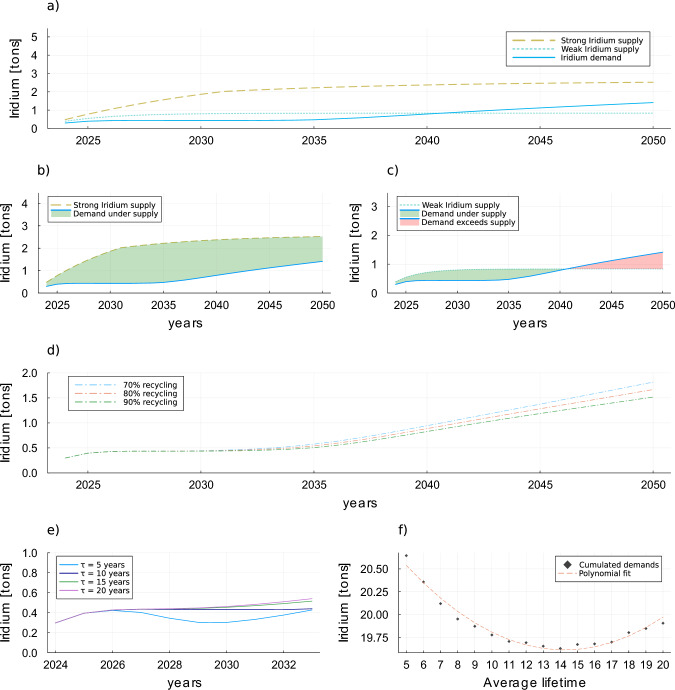
**The optimistic BAU scenario:** Projected iridium demands with an average lifetime τ=10 years and recycling efficiency of 97% by 2035 and corresponding supply projections. **a)** Demand and supply projections. Values from 2024-2030 reflect demands from planned capacity expansions. **b)** Supply to demand gaps under strong supply. **c)** Supply to demand gaps under weak supply. Red areas indicate time ranges in which demand is higher than supply whereas green areas indicate time ranges where supply is higher than demand. **d)** Iridium demands from variations of recycling rates γ for 70%, 80% and 90% reached by 2035. **e)** Iridium demands within the first 9 years from variations of the average lifetime of 5, 10, 15 and 20 years. **f)** Cumulative demands for the whole time range from lifetime variations ranging from 5 to 20 years. The underlying data for each figure are available in Supplementary Information SI 2 (spreadsheet tab “BAUopt”)

Under *optimistic* iridium material intensity assumptions, the BAU scenario shows substantially improved supply-demand dynamics (Figure 4a). As shown in Figure 4b, demand remains fully covered under the strong supply scenario. Under the weak supply scenario, by contrast, projected iridium demand exceeds available supply only after 2041 (Figure 4c). The resulting cumulative shortfall (red area) amounts to 3.1 tons, whereas the potential cumulative surplus (green area) reaches 4.6 tons, indicating that stockpiling during surplus years would be sufficient to meet the projected capacity expansion targets. Sensitivity analysis reveals that variations in the recycling rate (Figure 4d) have negligible impact throughout the time horizon, suggesting that a 70% recycling efficiency is sufficient, which is probably already achieved today (Clapp & Zalitis, 2023). Additionally, lifetime variations indicate iridium demand reductions in the first years of capacity expansions (Figure 4e) and that an average lifetime of τ=14 years minimizes cumulative demand (Figure 4f), offering additional potential for material savings. Overall, the *optimistic* BAU scenario appears achievable under current recycling rates and a range of different electrolyzer lifetimes.

### The conservative IEA-NZE scenario

**Fig. 5:**
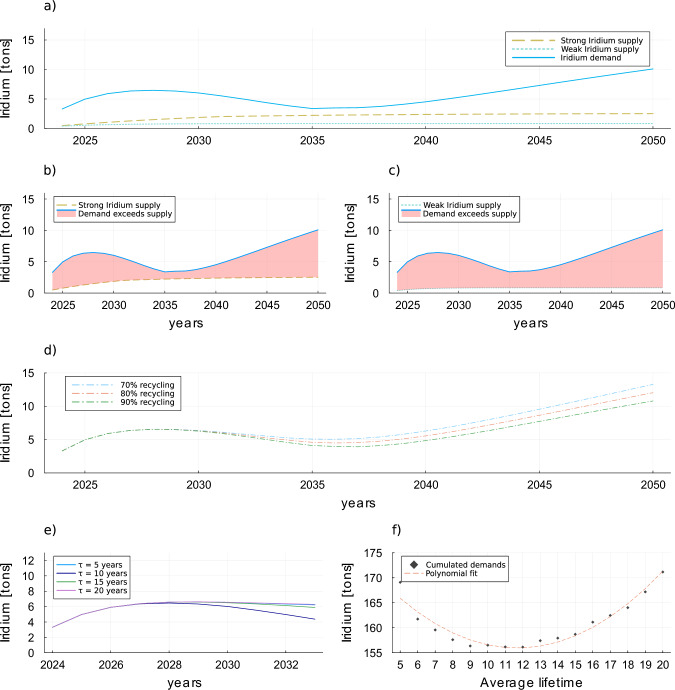
**The conservative IEA-NZE scenario:** Projected iridium demands with an average lifetime τ=10 years and recycling efficiency of 97% by 2035 and corresponding supply projections. **a)** Demand and supply projections. **b)** Supply to demand gaps under strong supply. **c)** Supply to demand gaps under weak supply. Red areas indicate time ranges in which demand is higher than supply whereas green areas indicate time ranges where supply is higher than demand. **d)** Iridium demands from variations of recycling rates γ for 70%, 80% and 90% reached by 2035. **e)** Iridium demands within the first 9 years from variations of the average lifetime of 5, 10, 15 and 20 years. **f)** Cumulative demands for the whole time range from lifetime variations ranging from 5 to 20 years. The underlying data for each figure are available in Supplementary Information SI 2 (spreadsheet tab "NZEcon")

Compared with the BAU scenario, the IEA-NZE scenario implies substantially more ambitious capacity expansion targets (Figure 2a), resulting in markedly higher projected iridium demand (Figure 5a). Even under the strong supply projection (Figure 5b), demand exceeds available supply throughout the analysis period; the same holds under the weak supply projection (Figure 5c).

Bridging the supply gap in the *conservative* IEA-NZE scenario would require approximately 101 tons of additional iridium under the strong supply projection and around 135 tons under the weak supply projection. This corresponds to an average increase in primary iridium production of 49% and 66%, respectively. Sensitivity analyses of recycling rates (Figure 5d) and average lifetime assumptions for 2024-2033 (Figure 5e) indicate that the magnitude of the shortfall remains largely unchanged, even with a shortfall-minimizing lifetime of τ=11-12 years (Figure 5f). These results suggest that the *conservative* IEA-NZE scenario is not feasible if annual primary iridium production remains fixed at approximately 7.5 tons.

### The optimistic IEA-NZE scenario

**Fig. 6:**
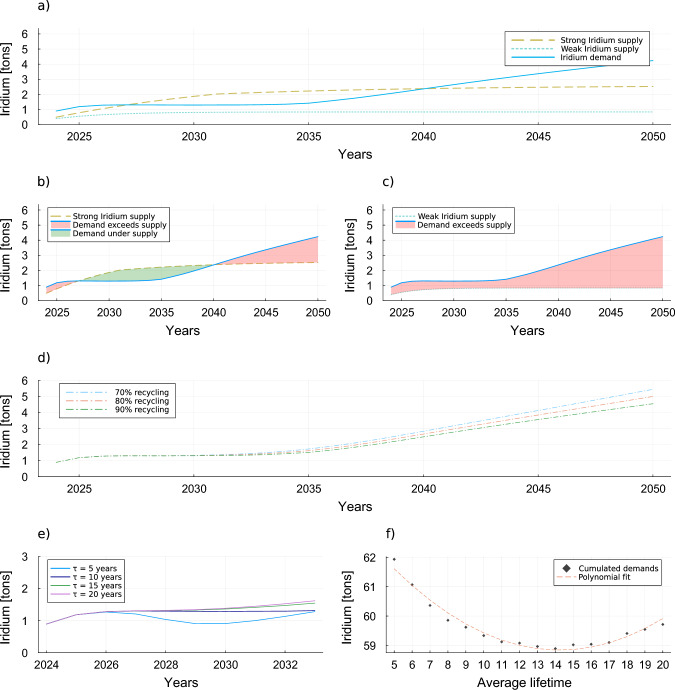
**The optimistic IEA-NZE scenario:** Projected iridium demands with an average lifetime τ=10 years and recycling efficiency of 97% by 2035 and corresponding supply projections. **a)** Demand and supply projections. **b)** Supply to demand gaps under strong supply. **c)** Supply to demand gaps under weak supply. Red areas indicate time ranges in which demand is higher than supply whereas green areas indicate time ranges where supply is higher than demand. **d)** Iridium demands from variations of recycling rates γ for 70%, 80% and 90% reached by 2035. **e)** Iridium demands within the first 9 years from variations of the average lifetime of 5, 10, 15 and 20 years. **f)** Cumulative demands for the whole time range from lifetime variations ranging from 5 to 20 years. The underlying data for each figure are available in Supplementary Information SI 2 (spreadsheet tab “NZEopt”)

Projected iridium demand in the *optimistic* IEA-NZE scenario is significantly lower than in the *conservative* case (Figure 6a). When compared to the strong supply projection, demand remains below supply from 2028 to 2040 (green area in Figure 6b), with supply deficits occurring during 2024-2027 and again from 2040-2050 (red areas). The initial shortfall of 1.07 tons during 2024-2027 could likely be covered by existing iridium stocks, which can be estimated to be at least 1 ton based on Johnson Matthey data (Brown et al., 2023). Between 2028 and 2040, a surplus of 6.9 tons could be accumulated, which would help offset the 9.7-ton deficit projected for 2040-2050, reducing the net gap to just 2.7 tons. In contrast, comparison to weak supply projections leads to supply shortages at any time (Figure 6c).

In contrast to the optimistic BAU scenario, the speed at which recycling efficiency ramps up plays a significant role in the realization of the optimistic IEA-NZE scenario. Figure 6a represents the baseline optimistic IEA-NZE case, in which the recycling efficiency increases linearly from 70% today to 97% by 2035. Figure 6d shows an otherwise identical scenario in which the recycling rate remains constant at 70% throughout the entire period. Comparing these two cases isolates the effect of recycling ramp-up alone and reveals a cumulative difference in iridium demand of 9.2 tons by 2050. This highlights that, under the ambitious deployment rates of the IEA-NZE scenario, rapid improvements in recycling efficiency become a decisive factor in mitigating primary iridium demand.

**Fig. 7:**
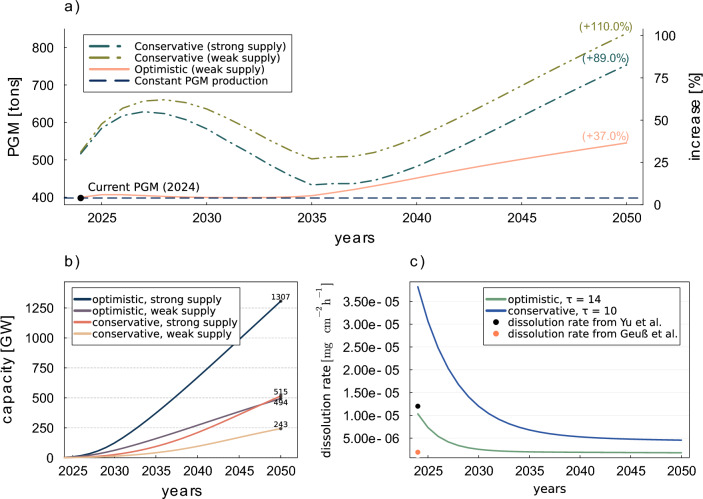
Required platinum group metal (PGM) production increase: Graph showing the required increase in PGM production in order to achieve sufficient iridium supply for the IEA-NZE scenario combinations listed in the legend. **b)** Maximum reachable capacities under constrained of iridium material intensities and supply projections. **c)** Required evolution of dissolution rates for average lifetime τ=10 and τ=14 alongside dissolution rate estimates derived from (Yu et al., 2020) and (Geuß et al., 2024). The underlying data for each figure are available in Supplementary Information SI 2 (spreadsheet tab “Discussion”)

Regarding the variation of lifetimes, similar to the previous scenarios, sensitivities show that lower lifetimes τ lead to a decrease in iridium demand in the first years of capacity expansion (Figure 6e). However, lower lifetimes again increase cumulative iridium demand over the full time horizon (Figure 6f), as in the previous scenarios.

Since for the strong supply estimate, initial demands within the years 2024 to 2027 should not pose a problem due to the probable existence of sufficient iridium stocks (see chapter 2.2), exploitation of this effect is unnecessary. Calculations even suggest, that a lifetime of τ=14 would lead to a minimization of cumulated iridium demands.

## Discussion

Under the BAU Scenario, capacity expansion targets are only at risk when both weak iridium supply projections and *conservative* iridium material intensity trajectories are assumed. In all other variations, the long-term targets appear achievable under average conditions. Notably, if iridium material intensities improve optimistically and strong supply projections hold, a recycling efficiency of just γ=0.7 is sufficient to meet projected iridium demand. In contrast, realizing the IEA-NZE scenario requires a more favorable combination: *optimistic* reductions in iridium material intensity, strong iridium supply, and near-maximum recycling efficiency of 97% by 2035. Even in the *optimistic* case, a shortfall of 3.8 tons may arise, but this could potentially be covered by reallocating recycled iridium from other sectors or by utilizing potentially existing stockpiles.

All other scenario combinations would require an increase in primary iridium production. However, achieving this is far from straightforward. As previously noted, iridium is mined exclusively as a byproduct of other platinum group metals (PGMs) (Brown et al., 2023; Clapp & Zalitis, 2023), meaning its supply is governed by the demand for platinum and palladium, not iridium itself. To estimate the scale of required PGM production increases, it is assumed that iridium constitutes approximately 2% of total PGM output (United States Geological Survey, 2023; Riedmayer et al., 2023). Based on this ratio, the additional PGM extraction needed to meet iridium demand under the most challenging IEA-NZE scenarios is shown in Figure 7a.

Estimates indicate that in both *conservative* scenarios, PGM production would need to increase by at least 25% as early as 2024 to generate sufficient iridium to meet projected demand. In the *optimistic* scenario, a more gradual increase starting around 2035 would be required. However, it remains uncertain whether primary PGM production will grow at all. For example, approximately 40% of mined platinum is currently used in automotive catalytic converters (Market News, 2023), a demand segment expected to decline with the ongoing electrification of vehicle fleets.

Conversely, several studies suggest that overall platinum demand may continue to rise due to its broad range of applications, including jewelry, glass manufacturing, electrolysis, and fuel cells (Brown et al., 2023; Aucutos Metal Portfolios, 202s). In particular, the latter is expected to play a critical role in sustaining PGM production, highlighting the interdependence of different platinum group metals. Reverdiau et al. (2021) estimate that platinum demand from PEMEL fuel cells alone could reach 1,000 tons annually by 2050, which would correspond to more than a five-fold increase compared to 2024 (World Platinum Investment Council, 2025). Under such conditions, the IEA-NZE targets could remain achievable, provided iridium material intensities improve optimistically. However, the *conservative* IEA-NZE scenarios would still be unattainable, as their initial material demands exceed feasible supply under any circumstance. This is further illustrated in Figure 7b, which shows maximum achievable capacity expansion under both *conservative* and *optimistic* assumptions for iridium material intensity and supply, based on Equation (5).

5 $$\begin{aligned} P_{el}^{i} = \frac{\omega ^i}{m_{total}^i} \end{aligned}$$

Thus, iridium material intensity $\omega ^i$ emerges as the most critical factor influencing iridium demand from PEMEL electrolysis. As previously discussed, literature values for current $\omega ^i$ vary significantly (Clapp & Zalitis, 2023). Eikeng et al. report a state-of-the-art value of 750 $[kg \cdot GW^{-1}]$, aligning with the starting point of the *conservative* scenario (Eikeng et al., 2024). If this value reflects current practice, achieving the IEA-NZE targets would require a rapid and substantial reduction in $\omega ^i$ by at least a factor of three to approximately 250 $[kg \cdot GW^{-1}]$. Several studies indicate that reductions of up to 90% relative to current systems may be technically feasible, comparable to historical platinum loading reductions achieved in PEM fuel cells (Clapp & Zalitis, 2023; Eikeng et al., 2024; Kiemel et al., 2021; Kongkanand & Mathias, 2016). Such reductions would correspond to advanced iridium intensities on the order of 50-100 $[kg \cdot GW^{-1}]$, provided that catalyst utilization and long-term stability can be maintained. In this context, achieving material intensities of approximately 250 $[kg \cdot GW^{-1}]$ by 2030 represents a conservative intermediate milestone rather than an ultimate lower bound. The decisive challenge, however, lies in the speed at which these reductions can be realized, as delayed improvements would substantially constrain the feasibility of ambitious deployment pathways such as the IEA-NZE scenario.

As highlighted by Clapp et al., reducing dissolution rates which quantifies the degradation of the iridium at the anode (measured in $mg \cdot cm^2 \cdot h^{-1}$) is essential for lowering iridium loadings while maintaining sufficient lifetimes (Clapp & Zalitis, 2023). Figure 7c presents the required dissolution rates to sustain 10- and 14-year electrolyzer lifetimes under both *conservative* and *optimistic* $\omega ^i$ trajectories. The dissolution rate reported by Yu et al. is included as a conservative benchmark, as it is derived from accelerated durability testing under dynamic operation and ultra-low iridium loadings, representing a stress-tested upper bound rather than intrinsic catalyst stability (Yu et al., 2020). In addition, Figure 7c includes a dissolution rate derived from recent results by Geuß et al., who demonstrate that rutile $IrO_2$ catalysts with loadings of approximately 0.1$mg\cdot cm^{-2}$ can sustain lifetimes of around 10 years (Geuß et al., 2024). Translating this reported loading-lifetime combination into an effective dissolution rate using the system-level assumptions of this study (see Supplementary Information SI 1 (Section S6)) yields values that fall below the dissolution rates required to sustain both 10- and 14-year lifetimes under the conservative and optimistic $\omega _i$ trajectories. This comparison indicates that, from a dissolution-rate perspective, current advances in rutile $IrO_2$ catalysts could already provide sufficient stability to support long PEM electrolyzer lifetimes at low iridium loadings. Consequently, further reductions in iridium-specific power density are not fundamentally limited by intrinsic catalyst dissolution, while remaining challenges primarily relate to the reliable transfer of such catalyst performance from laboratory conditions to industrial MEA and stack-level operation.

If iridium material intensities do not decline rapidly enough, one remaining, but questionable, strategy to reduce initial iridium demand would be to artificially shorten PEMEL lifetimes. As shown in the sensitivity analysis in Sect. 3, setting the average lifetime to τ=5 years can significantly lower primary iridium demand in the early years by accelerating entry into the recycling loop. For example, if iridium requirements per unit of capacity were reduced from 750 to 325 $[kg \cdot GW^{-1}]$ , the recycled iridium from older electrolyzers could nearly support the installation of twice as many new units. However, it remains uncertain whether such an approach would be economically viable without external support, such as targeted subsidies.

Beyond these economic uncertainties, several modeling limitations should be noted. The analysis assumes that all iridium entering the PEMEL system boundary is fully available for installation and end-of-life recovery, thereby neglecting potential losses during anode catalyst synthesis, membrane-electrode assembly fabrication, and stack manufacturing, and further assumes constant primary iridium supply as well as no structural growth in iridium demand from other sectors. In addition, projected improvements in recycling efficiency implicitly rely on sufficient economic incentives and timely deployment of recycling infrastructure, which may not materialize uniformly in practice.

## Conclusion

This study advances existing assessments of iridium demand for PEM water electrolysis by introducing a system-level, stock-flow-consistent modeling framework that explicitly captures electrolyzer vintages, stochastic lifetimes, end-of-life replacements, and competition for global iridium supply. In contrast to previous static or non-recursive approaches, this formulation reveals that long-term iridium demand and replacement requirements are systematically underestimated when feedbacks between deployment, degradation, and recycling are neglected. By jointly resolving technology deployment, material stocks, and recycling dynamics, the analysis provides an extension to existing basis for evaluating iridium supply bottlenecks under net-zero-aligned hydrogen deployment pathways. Building on this framework, the study quantifies future iridium demand from PEM electrolysis capacity expansions by integrating deployment targets, iridium material intensities, electrolyzer lifetimes, recycling rates, and supply projections. Two scenarios were formulated for capacity expansions, iridium material intensities, and iridium supplies each to capture a range of possible futures, and sensitivities based on lifetime and recycling variations were evaluated accordingly. While actual demand trajectories will likely fall between the bounds of the different scenarios, the analysis clearly shows that meeting ambitious capacity targets, such as IEA-NZE scenario requires a substantial and extremely rapid reduction in iridium material intensities, combined with either increased primary supply or highly efficient recycling. The results further demonstrate that the timing of reductions in iridium-specific power density is more critical than the ultimate minimum value achieved. Delayed improvements lead to irrecoverable supply bottlenecks during early deployment phases, even if very low iridium loadings become technically feasible at later stages: an effect that gets particularly pronounced when accounting for recursive end-of-life replacements. Under conservative assumptions, achieving a 40 % PEMEL market share appears infeasible, whereas such shares remain within reach under more optimistic trajectories (Figure 7). Explicitly accounting for recursive replacement demand highlights that these feasibility limits are more restrictive than suggested by non-recursive assessments. Beyond electrolyzer deployment choices alone, the present analysis shows that PEM electrolysis scalability is constrained by competition for global iridium supply across multiple sectors. Embedding PEM deployment within global iridium flows and competing market demands reveals that market dynamics can limit achievable PEMEL shares. Where such iridium bottlenecks restrict PEM deployment, alternative electrolysis technologies offer viable pathways. Anion Exchange Membrane (AEM) electrolysis and Alkaline Electrolysis (AEL) can serve applications where high dynamic response is not essential, enabling strategic technology diversification. Careful techno-economic matching between electrolysis technology and operational context can therefore reduce reliance on PEMEL and alleviate critical material constraints. Additional mitigation strategies include the development of novel catalysts, such as iridium-ruthenium alloys (Eikeng et al., 2024), and the recovery of iridium from PGM tailings (Gibson et al., 2023). Another avenue concerns the deliberate reduction of PEMEL lifetimes through lower catalyst loadings. While this would increase replacement frequency, it could reduce initial iridium demand and accelerate the entry of material into the recycling loop, potentially supporting faster capacity deployment under supply constraints. Advances in catalyst stability (Geuß et al., 2024) further indicate that intrinsic iridium dissolution could no longer be the primary limiting factor for long PEMEL lifetimes, shifting attention toward deployment speed, recycling scale-up, and system-level integration. Beyond the hydrogen sector, substitution of iridium in other industries represents an additional lever to relieve criticality pressures. Overall, this study underscores the urgency of integrating material availability into electrolysis deployment strategies and highlights the importance of combining technology choice, recycling infrastructure, and catalyst innovation to support sustainable hydrogen production at scale.

**Supporting Information SI1:** The supporting information provides additional details on the dynamic material flow model, system definition, comparison of recursive and non-recursive formulations, damped-trend forecasting of iridium prices and sectoral demand, scenario design, dissolutionrate calculation, model input parameters, and supplementary sensitivity plots supporting the analysis of iridium demand from global expansion of PEM electrolysis.

**Supporting Information SI2:** This supporting information excel spreadsheet provides data tables for the manuscript titled "Critical Iridium Demands Arising from Global Expansion of Proton Exchange Membrane Electrolysis".

## Supplementary Information

Below is the link to the electronic supplementary material.

Supplementary file 1 (pdf 1199 KB)

Supplementary file 2 (xlsx 187 KB)

## Data Availability

Data is available in the Supplementary Information Sheets. Model Code that generated the data can be given upon request.
