# Supplementary material for: Critical iridium demands arising from global expansion of proton exchange membrane electrolysis
Source: J Ind Ecol. 2026 May 18;30(4):1299–314. doi: 10.1007/s44498-026-00088-y (PMC13451499; doi:10.1007/s44498-026-00088-y)
Supplement: Supplementary file 1 [file 44498_2026_88_MOESM1_ESM.pdf]

SUPPORTING INFORMATION FOR:

Wortmann, B., Stolten D. & Heinrichs H.

Critical Iridium Demands Arising from Global Expansion of Proton Exchange Membrane Electrolysis

Journal of Industrial Ecology.

Summary

The supporting information provides additional details on the dynamic material flow model, system definition, comparison of recursive and non-recursive formulations, damped-trend forecasting of iridium prices and sectoral demand, scenario design, dissolution-rate calculation, model input parameters, and supplementary sensitivity plots supporting the analysis of iridium demand from global expansion of PEM electrolysis.

Contents

S1 Material Flow Model S-2

S2 System Definition S-4

S3 Comparison: Recursive and Non-Recursive Material Flow Models S-6

S4 Damped Trend Method for Price and Demand Forecasting S-9

S4.1 Model Equations S-9
S4.2 Model Features and Interpretation S-10
S4.3 Implementation S-10
S4.4 Estimating Sectoral Demand from Price Correlation S-11

S5 Scenario Overview S-14

S6 Calculation of Dissolution Rates S-16

S7 Complete Model and Scenario Input Data S-17

S8 Supplementary Plots S-18

Bibliography S-22

S1. Material Flow Model

Iridium demand projections are calculated using a dynamic material flow model. The algorithm used for the calculations is figuratively shown in figure S1. The material flow system is shown in Figure 1 of the main manuscript.

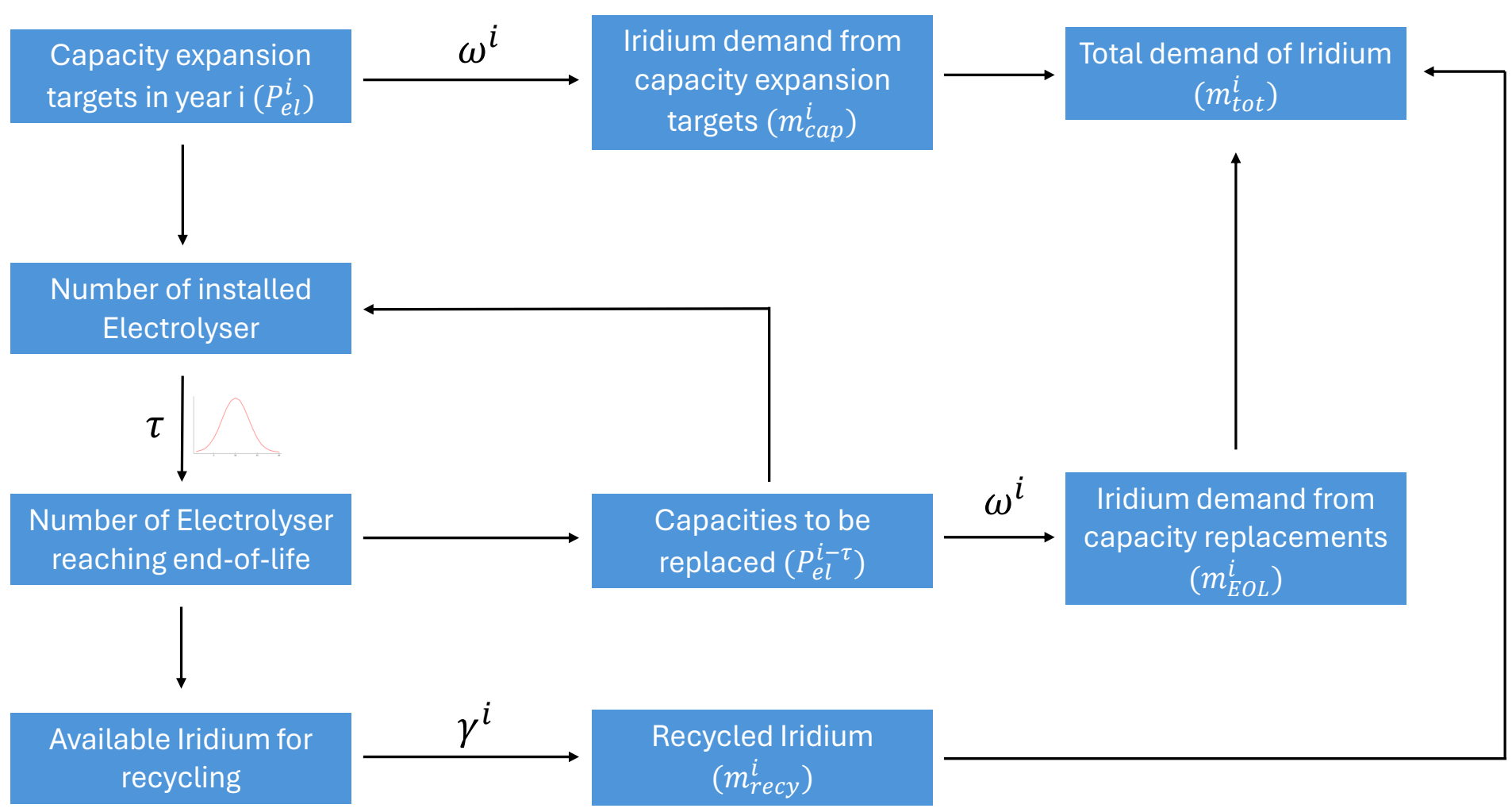

Figure S1: Computational algorithm for recursive iridium demand calculation. The flowchart shows how capacity expansion targets P_{el}^i are converted to iridium demand through material intensity ω^i, with end-of-life replacements feeding back into future demand calculations. Lifetime τ is sampled from a truncated normal distribution for each installed unit.

The iridium demand in year i, m^i_{total}, is calculated as

$$m^i_{\text{total}} = m^i_{\text{cap}} + m^i_{\text{EOL}} - m^i_{\text{recycling}} \tag{1}$$
$$m^i_{\text{cap}} = P^i_{\text{el}} \cdot \omega^i \tag{2}$$
$$m^i_{\text{EOL}} = P^{i-\tau}_{\text{el}} \cdot \omega^i \tag{3}$$
$$m^i_{\text{recycling}} = (m^{i-\tau}_{\text{cap}} + m^{i-\tau}_{\text{EOL}}) \cdot \gamma^i \tag{4}$$

where τ is the average electrolyzer lifetime, ω^i is the iridium-specific power density, and γ^i is the recycling efficiency. Lifetime is assigned to each installed

unit as $N(\tau, \sigma^2)$, where $\sigma = \tau/3$, bounded to $[1, 2\tau]$ (see Figure S2). Specific model parameter meanings can be read off table S2.

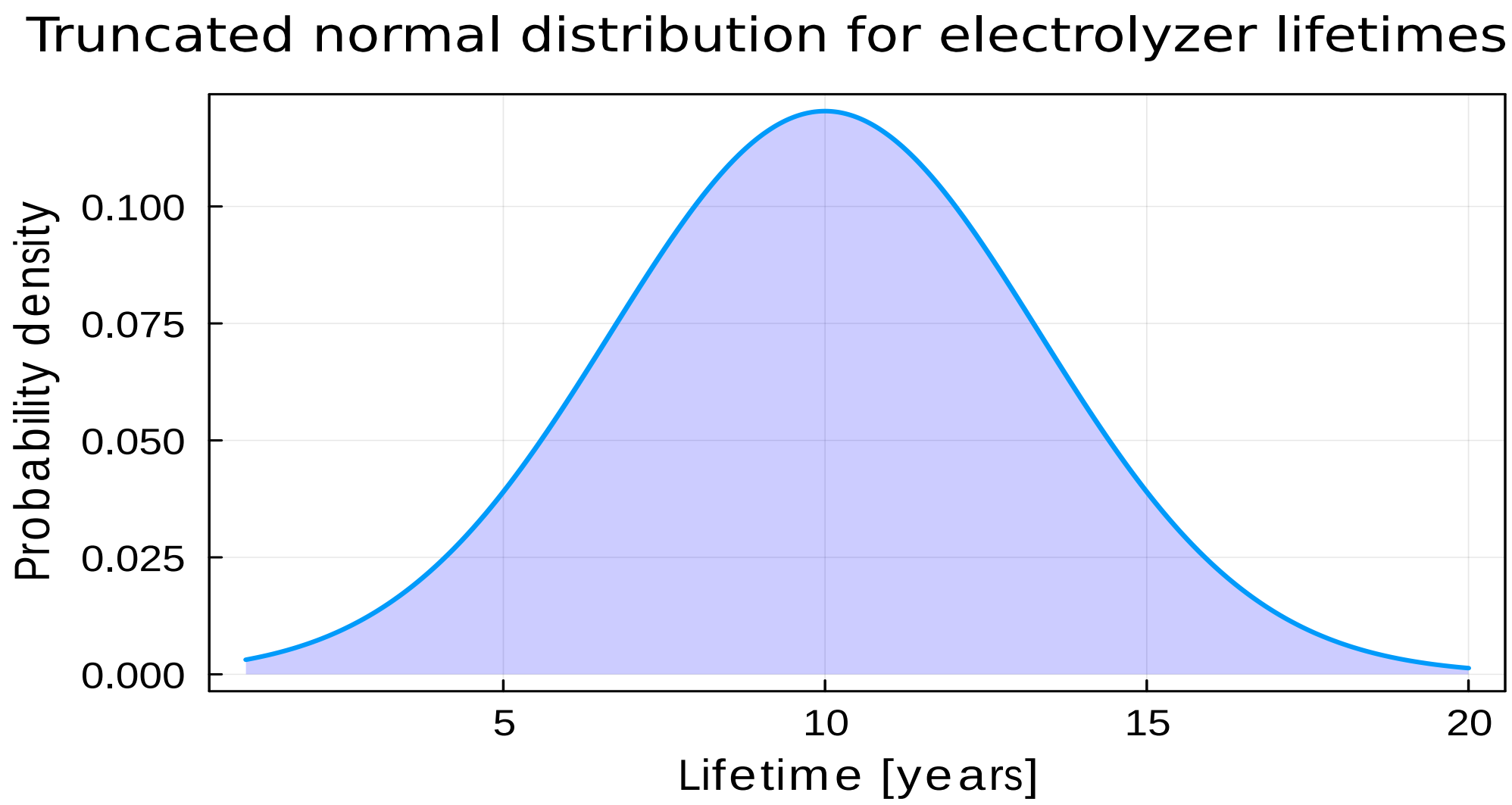

Figure S2: Truncated normal distribution for an average lifetime $\tau = 10$.

The algorithm for the calculation of iridium demand projections is schematically shown in Figure S1 and calculated following these steps:

1. For each year i from 2024 to 2050:
   (a) Calculate new PEMEL installations, P_{el}^{i}, based on scenario.
   (b) Assign individual lifetimes to new installations (see above).
   (c) Record the expected EOL year for each unit.
   (d) Sum replacements in year i from units installed in $i - \tau$.
   (e) Calculate total EOL demand and available recycled iridium.
   (f) Update stocks and flows for subsequent years.

S2. System Definition

The material flow system underlying the dynamic iridium demand model is defined in Figure 1 in the main manuscript and shown here again in Figure S3. Computational implementation follows the algorithm scheme in Figure S1. The system boundary comprises global iridium flows related to PEM electrolysis for the period 2024–2050. Processes and flows follow established material flow analysis (MFA) conventions, with stocks residing only in processes where material accumulation occurs. Table S1 summarizes all processes included in the system definition.

No.	Process Name	Description	Stock
1	Primary production	Global primary iridium mining and refining supplying market-grade iridium.	No
2	Iridium market pool	Allocation node distributing primary and secondary iridium to PEMEL manufacturing and competing markets.	No
3	PEMEL manufacturing	Manufacturing and commissioning of PEM electrolysis systems requiring iridium input.	No
4	In-use iridium in PEMEL	Iridium embedded in operating PEM electrolysis systems. The stock evolves dynamically according to $\Delta S_4(t) = F_{3\to4}(t) - F_{4\to5}(t)$.	Yes
5	End-of-life collection	Decommissioned PEMEL systems reaching end-of-life and entering the recycling stage.	No
6	Recycling	Recovery and refining of iridium from end-of-life PEMEL systems with efficiency γ_i.	No

Table S1: Process definitions for the material flow system shown in Figure S3.

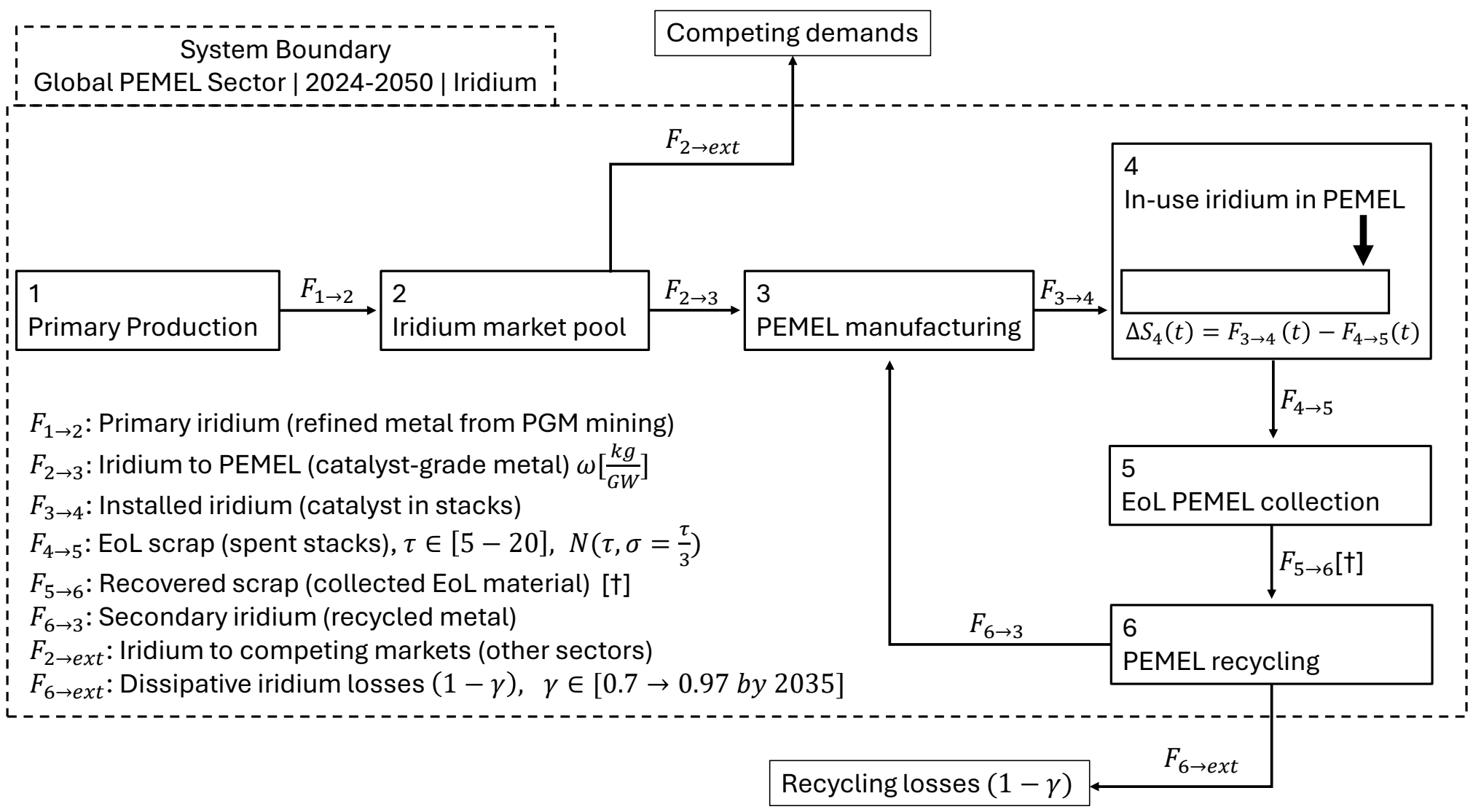

Figure S3: Dynamic material flow model for iridium in the global PEMEL sector (2024–2050). Numbered boxes represent system processes; labeled arrows represent iridium mass flows between processes, indexed by origin and destination process number. The stock change in process 4 (in-use iridium) is defined as $\Delta S_4(t) = F_{3\to 4}(t) - F_{4\to 5}(t)$. Flows exiting the system boundary include iridium allocated to competing markets ($F_{2\to \mathrm{ext}}$, exiting at process 2) and dissipative recycling losses ($F_{6\to \mathrm{ext}}$, exiting at process 6), given by $(1-\gamma)\cdot F_{5\to 6}$. Primary iridium supply is assumed constant at 7.5 t yr^{-1}. † The recovery parameter γ is applied as a single aggregated factor spanning both EoL collection (P5) and recycling processing (P6), i.e. $\gamma = \gamma_{\mathrm{col}} \cdot \gamma_{\mathrm{rec}}$, and the model does not resolve these two efficiencies separately (see Eq. 4). γ is assumed to increase linearly from 0.70 to 0.97 by 2035. Consequently, the scrap stream $F_{5\to 6}$ represents an aggregated EoL material flow rather than an explicitly tracked scrap commodity; manufacturing scrap and iridium losses during MEA and stack fabrication are not resolved as separate flows and are assumed negligible relative to the EoL scrap volumes over the modeled horizon.

S3. Comparison: Recursive and Non-Recursive Material Flow Models

This section directly addresses the methodological difference between recursive and non-recursive material flow modeling approaches in the context of iridium demand for PEM electrolysis. To isolate the effect of recursive end-of-life (EoL) replacement dynamics, we reproduce the non-recursive model presented by Clapp et al. (Clapp et al., 2023) and compare it against an otherwise identical recursive formulation. All comparisons hold the same techno-economic assumptions unless explicitly stated otherwise. We first reproduce the results reported by Clapp et al. using their published assumptions on capacity expansion, lifetime distributions, recycling rates, and iridium-specific power density. Figure S2-1 overlays the original results from Clapp et al. with outputs from our independently implemented non-recursive model (see Figure S4).

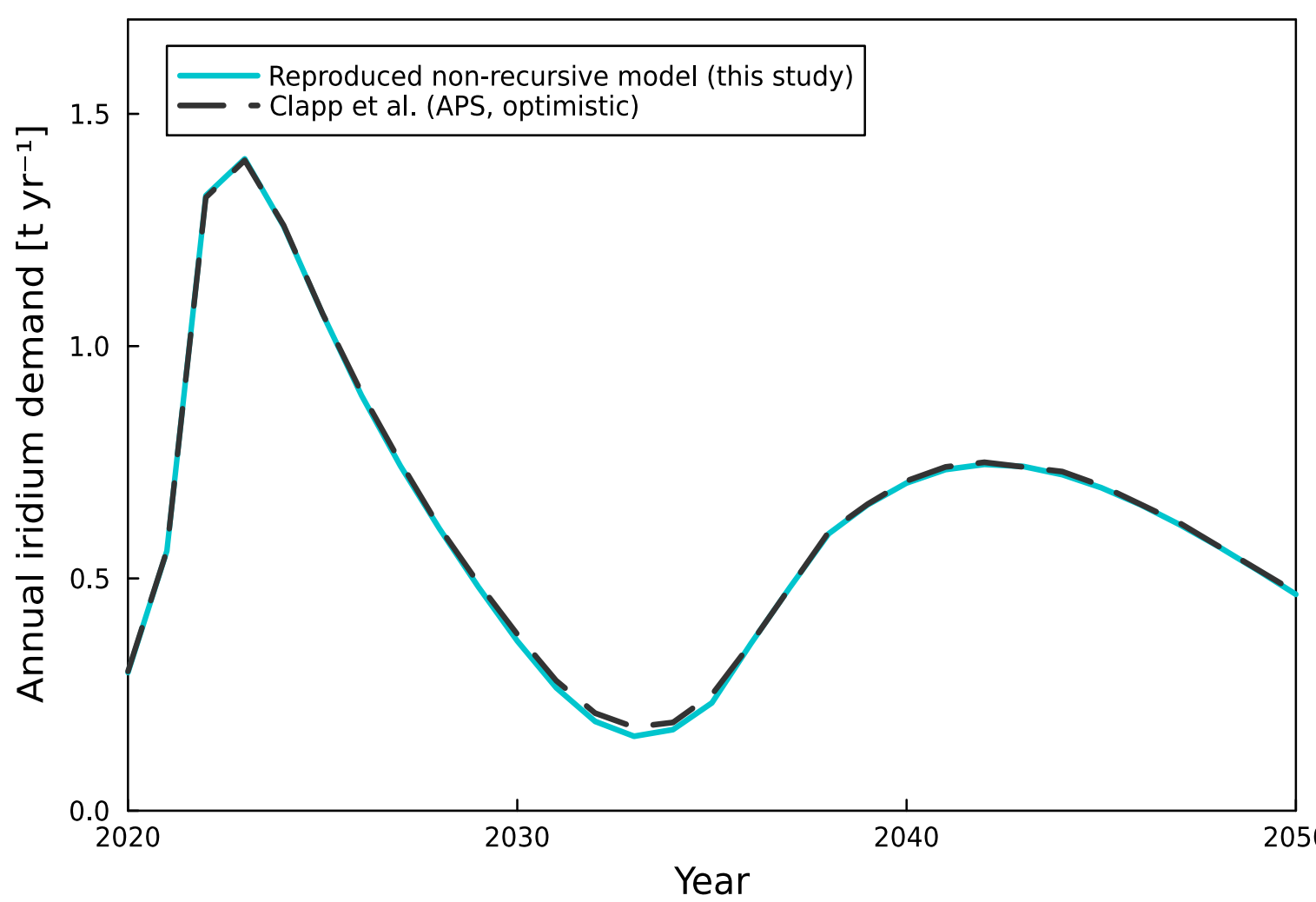

Figure S4: Reproduction of annual iridium demand in the optimistic APS scenario from Clapp et al. (Clapp et al., 2023). The reproduced non-recursive model closely matches the original results under identical assumptions, confirming consistency between implementations within a reasonable numerical error margin.

Having established that the non-recursive implementation used in this study faithfully reproduces the results of Clapp et al. (Clapp et al., 2023) under identical assumptions, we now isolate the effect of recursive end-of-life replacement dynamics. To this end, we compare the reproduced non-recursive

model directly with the recursive formulation developed in this study, while keeping all input parameters unchanged. As an illustrative example, we focus on the optimistic IEA-NZE scenario, which in the non-recursive framework reaches apparent iridium self-sufficiency i.e., zero primary iridium demand during the later stages of capacity expansion. This scenario provides a particularly transparent test case to assess how recursive treatment of replacement demand alters long-term iridium requirements. Figure S5 illustrates how recursive treatment of end-of-life replacement dynamics alters iridium demand trajectories under otherwise identical assumptions. In the optimistic IEA-NZE scenario, the non-recursive formulation reproduces the apparent iridium self-sufficiency reported by Clapp et al., with primary demand declining to zero in the later years of capacity expansion (Figure S5a). However, once replacement demand is treated recursively, primary iridium requirements remain non-zero despite high recycling rates. As shown in Figures S5b and S5c, this divergence arises because replacement-driven capacity additions feed back into future material demand in the recursive formulation, whereas they remain decoupled from stock evolution in the non-recursive case. The shaded areas highlight the cumulative impact of this feedback, demonstrating that neglecting recursive replacement dynamics leads to systematically lower demand and recycling.

While the optimistic IEA-NZE scenario provides a transparent illustration of the mechanisms introduced by recursive replacement dynamics, the observed differences are not limited to a single parameter set. We therefore extend the comparison to the full range of lifetime and recycling rate variations explored in this study, directly contrasting recursive and non-recursive formulations under otherwise identical assumptions. This allows us to assess the robustness and magnitude of recursion-induced deviations across a broader parameter space.

Figure S6 demonstrates that the divergence between recursive and non-recursive formulations persists across a broad parameter space and is strongly modulated by lifetime and recycling assumptions. Over the full modeling horizon (Figures S6a,b), recursive treatment leads to consistently higher cumulative primary demand and recycling flows, with the largest deviations occurring for short lifetimes and high recycling efficiencies. When restricting the analysis to the final 15 years of capacity expansion (Figures S6c,d), these differences become substantially more pronounced, with amplification factors exceeding unity by several multiples for primary demand. This indicates that non-recursive formulations increasingly underestimate material

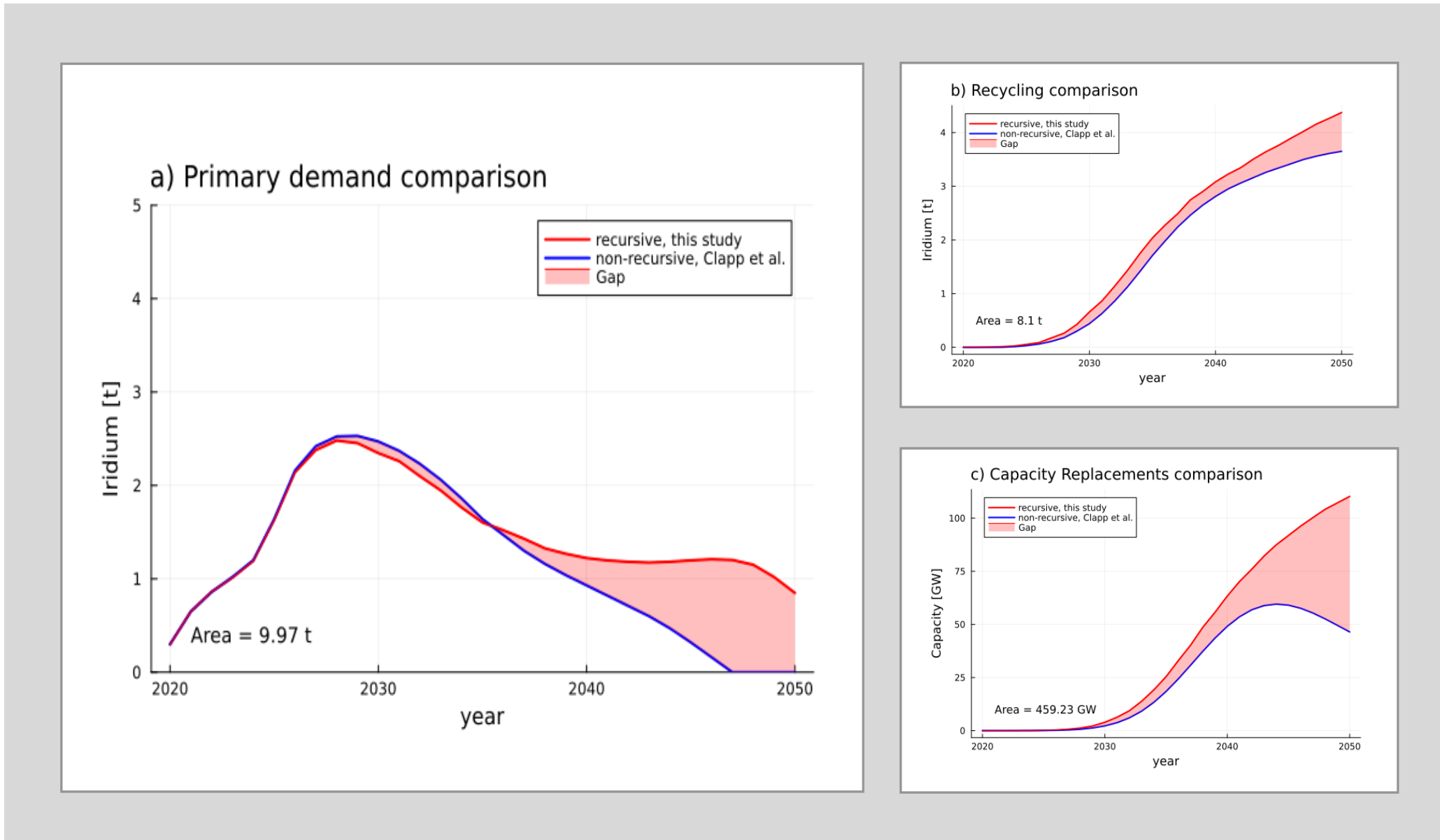

Figure S5: Comparison of recursive and non-recursive material flow formulations under identical input assumptions for the optimistic IEA-NZE scenario adopted from Clapp et al. (Clapp et al., 2023). Panels show (a) annual primary iridium demand, (b) secondary iridium supply from recycling, and (c) capacity additions driven by replacement of end-of-life PEM electrolysis units. The non-recursive formulation reproduces the apparent iridium self-sufficiency reported by Clapp et al., with primary demand declining to zero in later years. In contrast, the recursive formulation captures the feedback of replacement demand into future stock evolution, resulting in sustained primary iridium requirements. Shaded areas indicate the cumulative difference between both formulations, highlighting the integrated impact of recursive replacement dynamics over time. The underlying data for each figure are available in SI spreadsheet "Model Comparison Data".

requirements precisely in phases where replacement-driven capacity additions dominate system dynamics, highlighting the relevance of recursive modeling for long-term, net-zero-aligned scenarios.

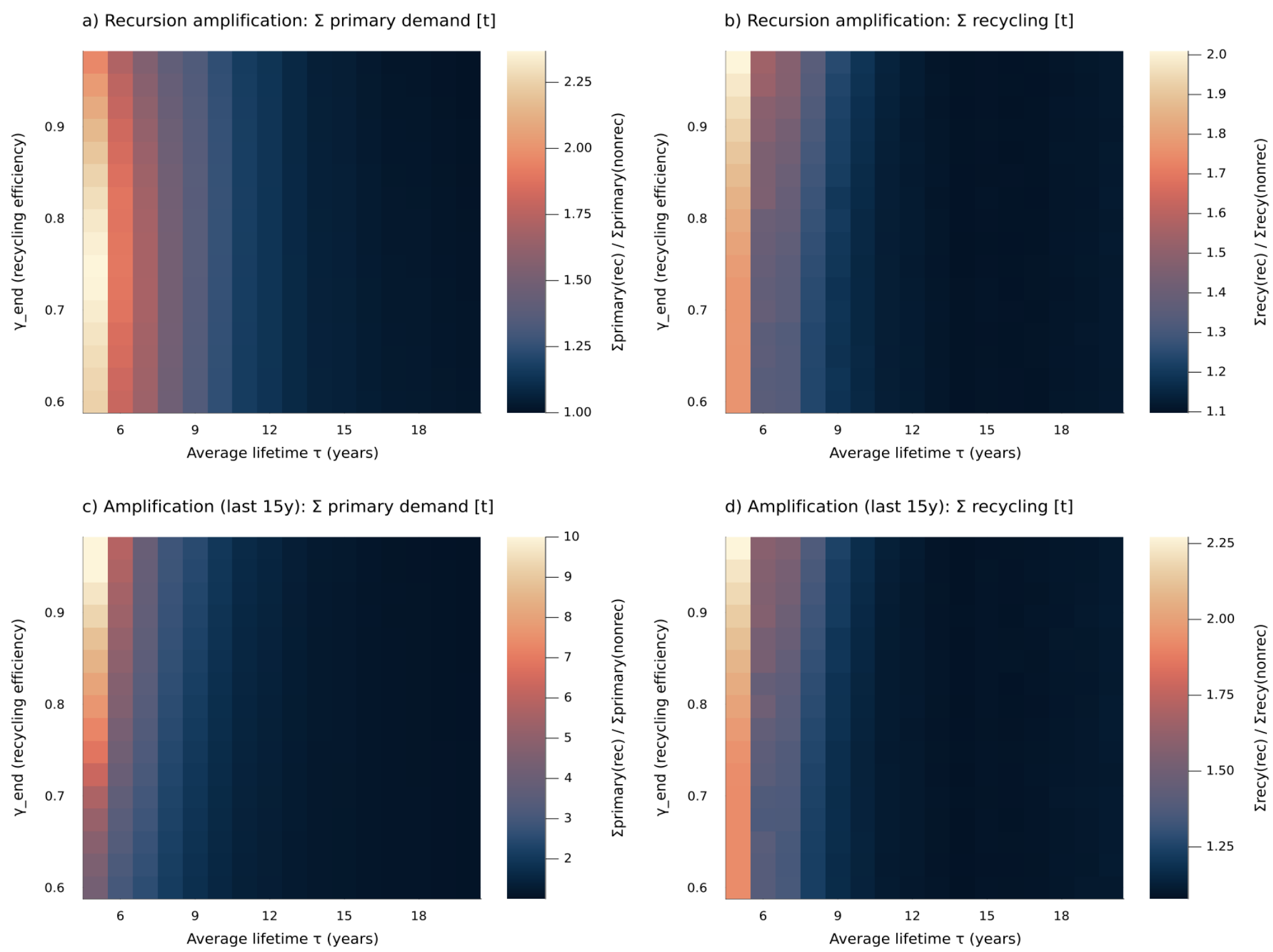

Figure S6: Systematic comparison of recursive and non-recursive model formulations across variations in average lifetime τ and end-of-life recycling efficiency γ_{end}. Panels (a) and (b) show the ratio between cumulative primary iridium demand and cumulative recycling, respectively, obtained from the recursive model relative to the non-recursive formulation over the full modeling horizon. Panels (c) and (d) report the same ratios restricted to the final 15 years of capacity expansion. Color values therefore indicate the amplification factor introduced by recursive treatment of replacement dynamics.

S4. Damped Trend Method for Price and Demand Forecasting

To project future iridium prices, we employ the damped trend exponential smoothing method, as described by Holt (Holt, 1957) and commonly used in time series analysis (Hyndman and Athanasopoulos, 2018; Cueva, 2023). This approach captures both the level and trend of the series while preventing the trend from diverging indefinitely, a common issue in standard Holt's linear method.

S4.1. Model Equations

The damped trend model uses three smoothing parameters:

- α (level smoothing)
- β (trend smoothing)
- ϕ (damping parameter, $0 < \phi < 1$)

The updating equations are:

$$\begin{aligned} \text{Level:} \quad & \ell_t = \alpha y_t + (1-\alpha)(\ell_{t-1} + \phi b_{t-1}) && (5) \\ \text{Trend:} \quad & b_t = \beta(\ell_t - \ell_{t-1}) + (1-\beta)\phi b_{t-1} && (6) \\ \text{Forecast:} \quad & \hat{y}_{t+h|t} = \ell_t + (\phi + \phi^2 + \cdots + \phi^h) b_t && (7) \end{aligned}$$

where:

- y_t is the observed value at time t
- ℓ_t is the estimated level at time t
- b_t is the estimated trend at time t
- $\hat{y}_{t+h|t}$ is the forecast h steps ahead from time t
- ϕ is the damping parameter ($0 < \phi < 1$); when $\phi = 1$ the method reduces to Holt's linear trend

S4.2. Model Features and Interpretation

- The damping parameter (ϕ) controls the extent to which the trend persists into the future. For $0 < \phi < 1$, the trend is "damped," causing forecasts to eventually plateau rather than grow linearly.
- Smoothing parameters α and β (chosen via cross-validation or maximum likelihood) control the weight given to recent observations versus previous estimates.
- This method is well-suited for material price series, which may exhibit trends that are not sustained indefinitely.

S4.3. Implementation

The model was implemented using algorithms available as provided by Cueva (Cueva, 2023) and validated following Hyndman (Hyndman and Athanasopoulos, 2018) and Athanasopoulos (Cueva, 2023). All parameters were fit to historical iridium price data.

S4.4. Estimating Sectoral Demand from Price Correlation

To estimate future iridium demand by sector, we analyze the historical correlation between iridium prices and sectoral demand, and use this relationship to project demand under various price forecast scenarios. For each sector s, we compute the Pearson correlation coefficient (r_s) between average annual iridium price (p_t) and demand ($d_{s,t}$) over the historical data period ($t = 1, \ldots, N$):

$$r_s = \frac{\sum_{t=1}^{N}(p_t - \bar{p})(d_{s,t} - \bar{d}_s)}{\sqrt{\sum_{t=1}^{N}(p_t - \bar{p})^2}\sqrt{\sum_{t=1}^{N}(d_{s,t} - \bar{d}_s)^2}} \tag{8}$$

where $\bar{p}$ and $\bar{d}_s$ are the mean price and mean demand in sector s over the historical period. Assuming a linear relationship between price and demand for each sector, we fit a linear regression model of the form:

$$d_{s,t} = \alpha_s + \beta_s p_t \tag{9}$$

where:

- $d_{s,t}$ is the demand in sector s at year t
- p_t is the iridium price at year t
- α_s is the sector-specific intercept
- β_s is the regression coefficient (slope), estimated from historical data

We estimate α_s and β_s using ordinary least squares (OLS) regression over the historical period. The strength of the price-demand relationship is indicated by the correlation coefficient r_s. To project future sectoral demand, we use the forecasted price $\hat{p}_t$ as obtained from the damped trend model as input into the regression (see Figure S7d):

$$\hat{d}_{s,t} = \alpha_s + \beta_s \hat{p}_t \tag{10}$$

where $\hat{d}_{s,t}$ is the projected demand in sector s for year t.

- The regression model assumes that the historical price-demand relationship remains valid in the forecast period.
- If sectoral demand is weakly correlated with price (low $|r_s|$), forecasts should be interpreted with caution.
- The approach enables scenario analysis by plugging in alternative price forecasts to estimate potential sectoral responses.

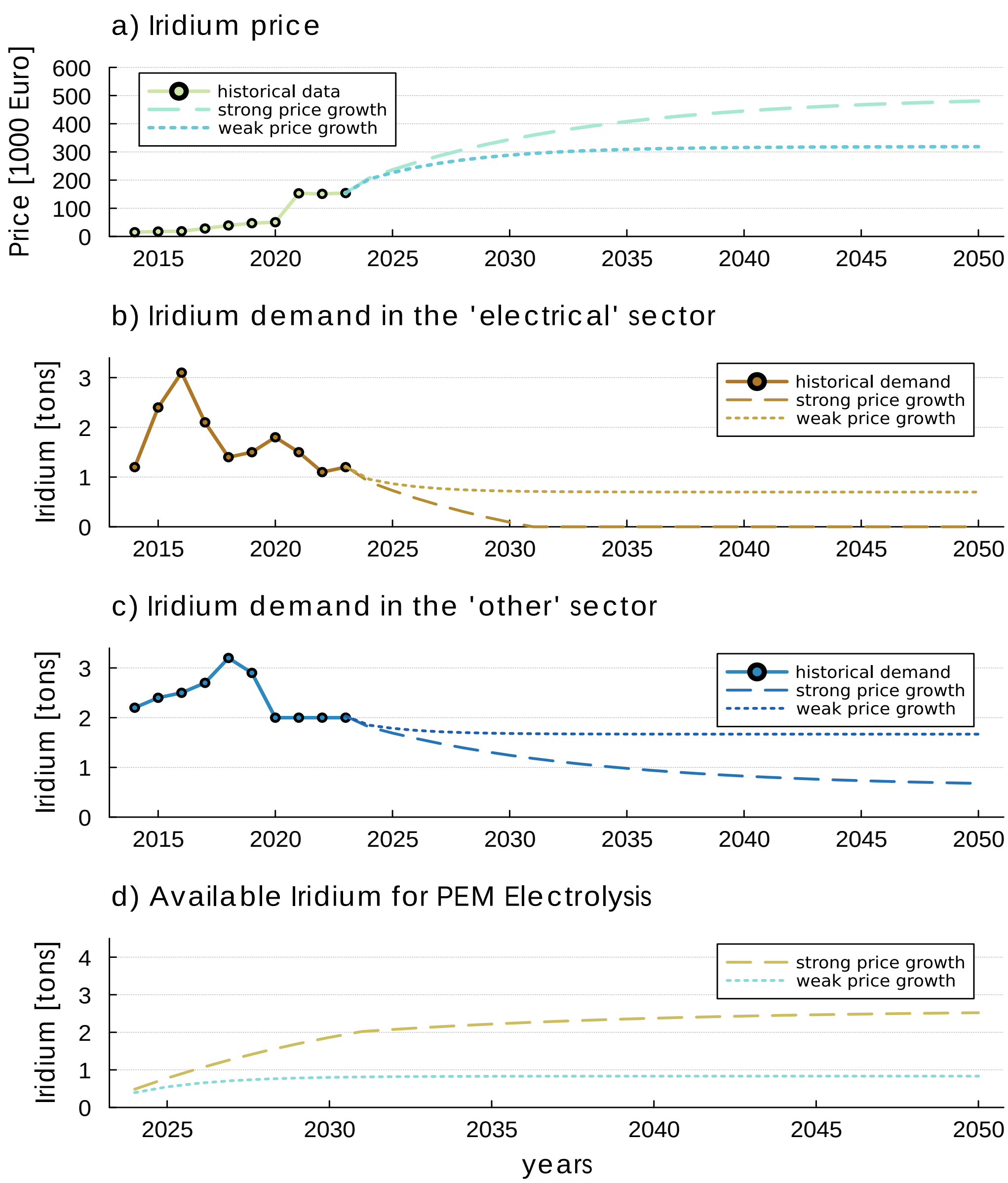

Figure S7: **a)** Historical price data and forecast up to 2050. **b)** Historical demand data and forecast for the 'electrical' sector. **c)** Historical demand data and forecast for the 'other' sector. **d)** Iridium supply projections from correlations with prices forecasts.

S5. Scenario Overview

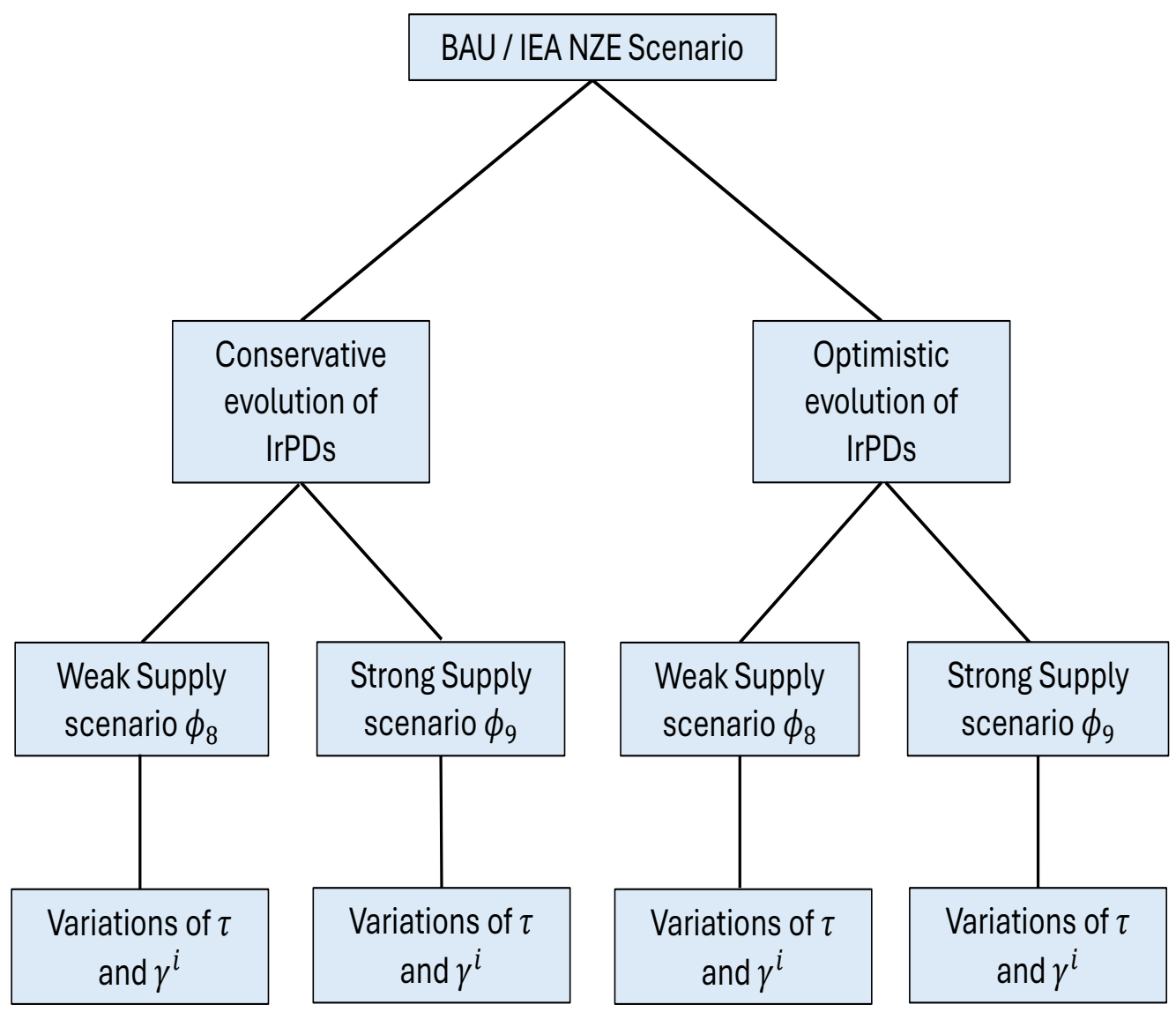

Figure S8: **Scenario overview:** Starting by one of the capacity expansion scenarios, calculations are continued for conservative and optimistic iridium-specific power densities (IrPDs) and compared to strong and weak supply scenarios, where ϕ_9 and ϕ_8 are the corresponding damping parameters. Upon there, sensitivity calculation for the recycling rate γ and average lifetime τ are performed.

To systematically explore future iridium requirements for PEM electrolysis under uncertainty, we developed a structured scenario set (see Figure S8). The analysis starts from two different capacity expansion scenarios (Business as usual and IEA-NZE scenario). From this baseline, we branch scenarios along three main axes:

1. **Technological Evolution:** Two trajectories for iridium-specific power density, a conservative evolution (slow improvement) and an optimistic evolution (rapid improvement) (Clapp et al., 2023).

2. **Supply Side Uncertainty:** For each IrPD trajectory, we consider two supply scenarios, defined by different values for the recycling rate (γ^i) and the share of secondary iridium (ϕ_8, ϕ_9).
3. **Parameter Sensitivities:** Within each supply scenario, we further explore the effect of variations in the average electrolyzer lifetime (τ) and recycling rate (γ^i).

S6. Calculation of Dissolution Rates

Dissolution rates d_r $[mg \cdot cm^{-2} \cdot h^{-1}]$ for according lifetimes τ $[years]$ and loading l_d $[mg \cdot cm^{-2}]$ where calculated following

$$d_r = \frac{l_d}{\tau \cdot 365 \cdot 24 \cdot l_f} \tag{11}$$

assuming a load factor l_f of 0.6 as in Clapp et al. (Clapp et al., 2023). Iridium specific power densities where converted to loading assuming constant current densities of 1.5 Acm^{-2} and decreasing cell voltages from $1.8V$ to $1.55V$.

S7. Complete Model and Scenario Input Data

An overview on the model input parameters and their ranges, as well as corresponding references is given by table S2.

**Parameter**	**Symbol / Unit**	**Default Value / Range**	**Source / Comment**
Iridium-specific power density	ω^i [kg GW^{-1}] or [mg W^{-1}]	250 (optimistic in 2024) 750 (conservative in 2024) kg GW^{-1}	Eikeng et al. (Eikeng et al., 2024), Clapp et al. (Clapp et al., 2023)
Electrolyzer life-time	τ [years]	10 (varied: 5, 10, 15, 20)	10 years base lifetime from (Clapp et al., 2023), variations are scenario parameter
Installed PEMEL capacity	P_{el} [GW]	2050 target 480 GW for BAU and 1468 for NZE	IEA-NZE (IEA, Paris, 2023), BAU (IEA, PAris)
Recycling efficiency	γ^i [–]	0.7 → 0.97 (2023–2050, ramped)	Literature; Johnson Matthey, Eikeng et al.
Dissolution rate	r_{diss} [mg cm^{-2} h^{-1}]	1×10^{-5} (varied)	Literature; Yu et al. (Yu et al., 2020)
Primary iridium supply	S^i_{prim} [tons]	7–10 tons/year (varies)	Johnson Matthey (Brown et al., 2023), USGS (Survey)

Table S2: Key parameters used in the material flow model with variations for sensitivities and scenarios alongside references.

S8. Supplementary Plots

The complete range of sensitivity results can be seen in the figures below. For each scenario combination the average lifetime τ is varied and demand as well as recycling projections shown as spread plots.

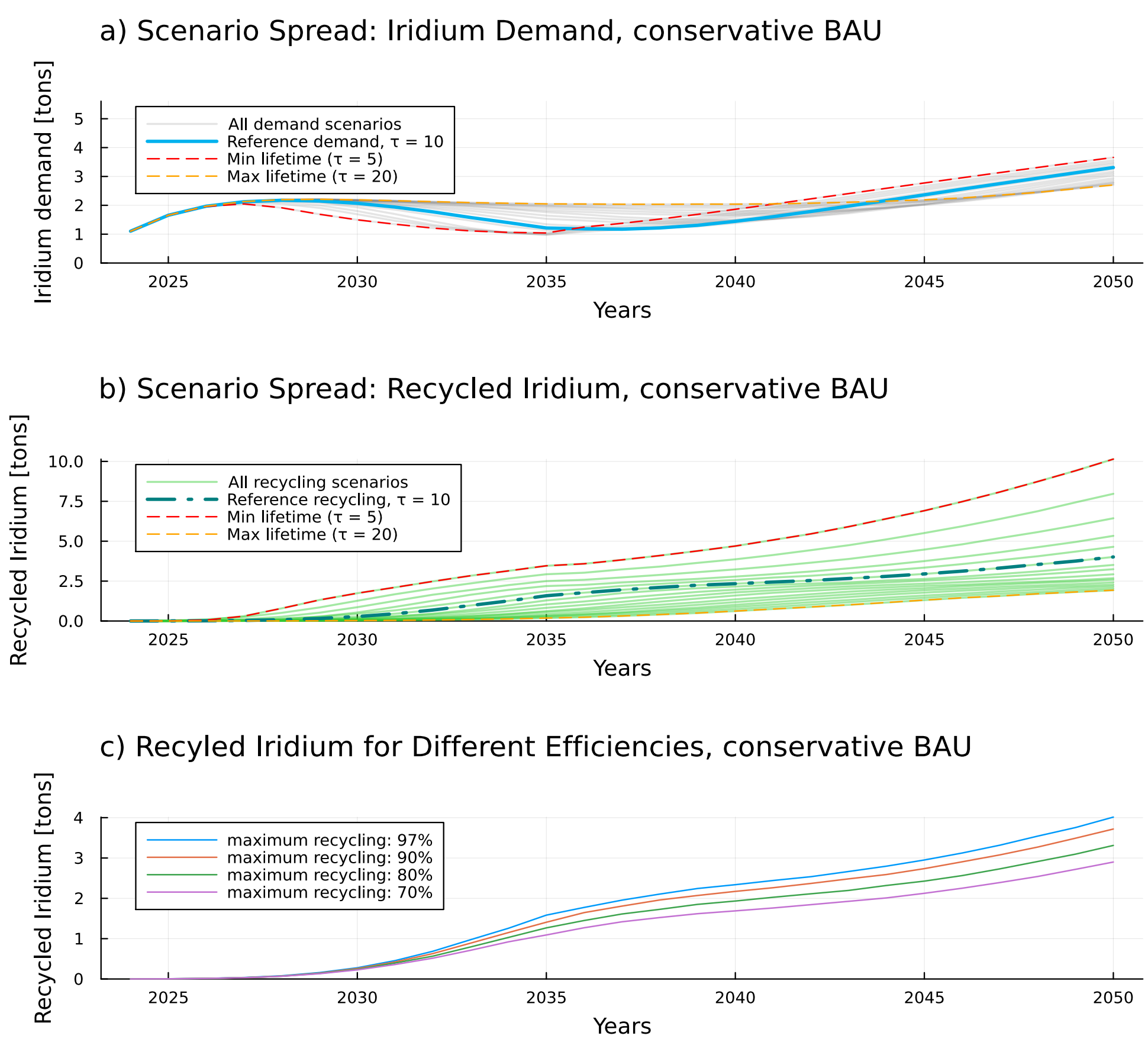

Figure S9: **Conservative BAU scenario spread:** a) Iridium demand range for varying lifetimes $\tau = 5 - 20$. b) Iridium recycling for varying lifetimes $\tau = 5 - 20$ calculated based on equation 4. c) Recycled annual iridium amounts for fixed lifetime $\tau = 10$ and target recycling rates of 97% (baseline scenario),90%,80%, and 70%.

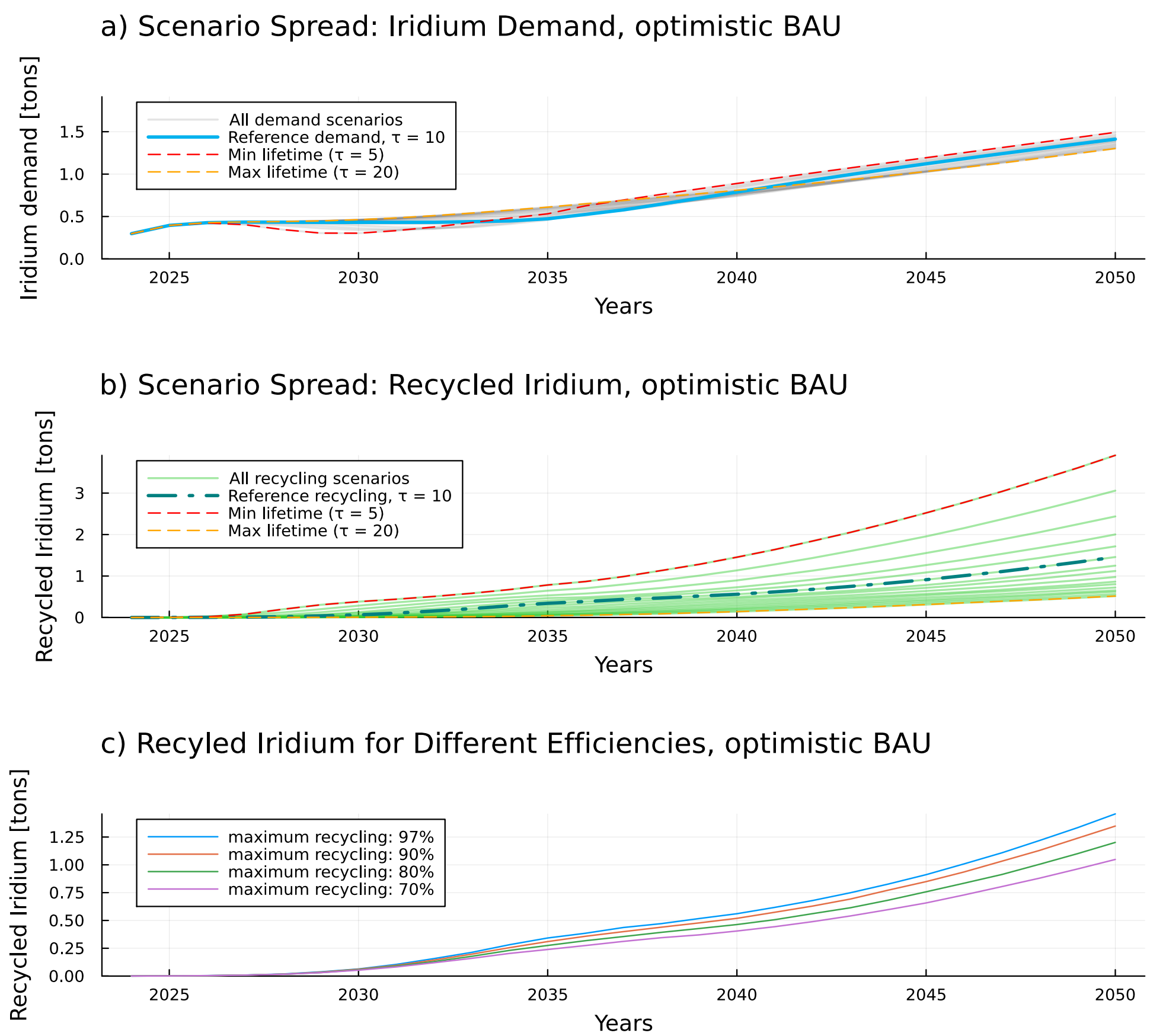

Figure S10: **Optimistic BAU scenario spread:** a) Iridium demand range for varying lifetimes $\tau = 5 - 20$. b) Iridium recycling for varying lifetimes $\tau = 5 - 20$ calculated based on equation 4. c) Recycled annual iridium amounts for fixed lifetime $\tau = 10$ and target recycling rates of 97% (baseline scenario),90%,80%, and 70%.

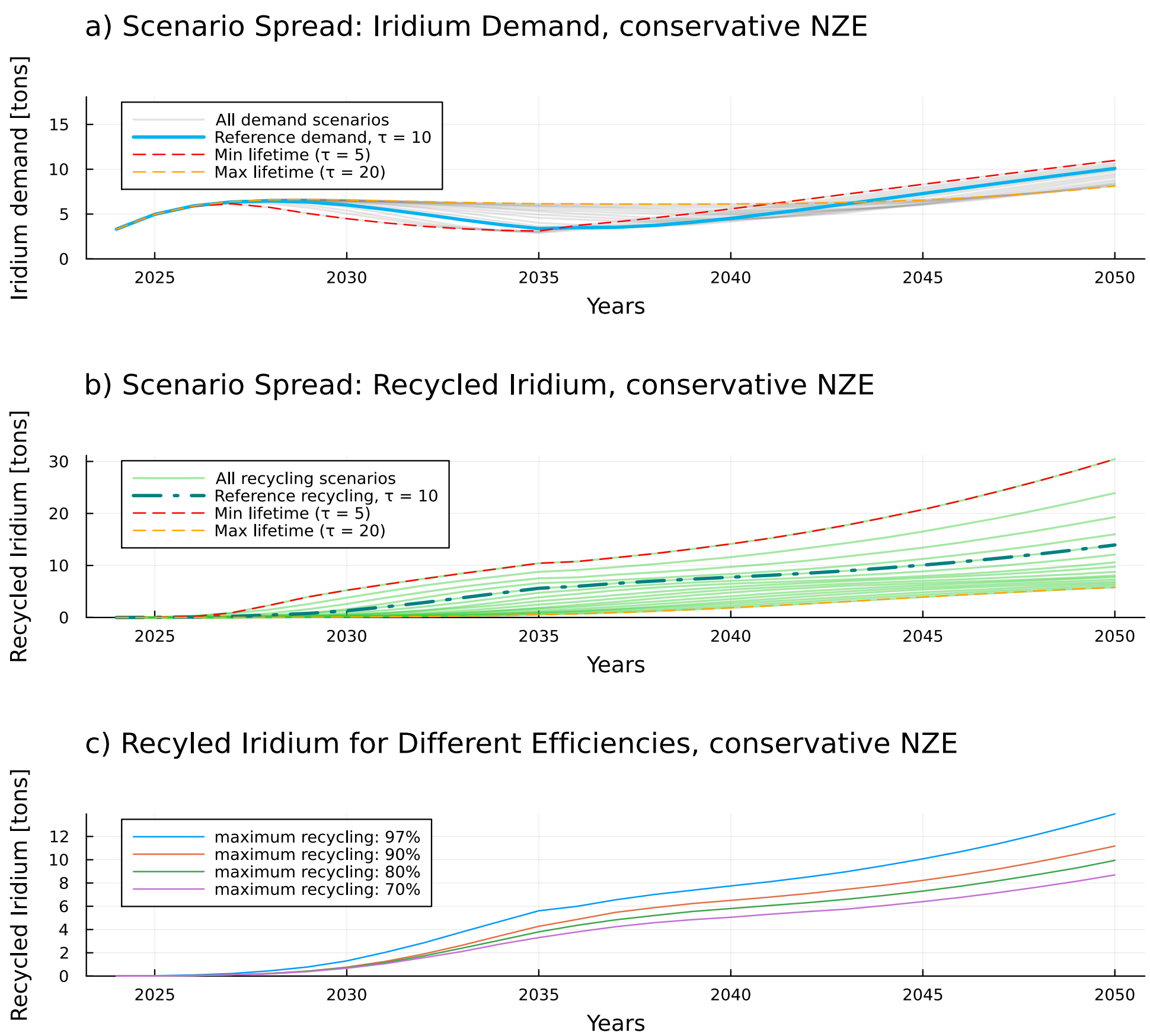

Figure S11: **Conservative NZE scenario spread:** a) Iridium demand range for varying lifetimes $\tau = 5 - 20$. b) Iridium recycling for varying lifetimes $\tau = 5 - 20$ calculated based on equation 4. c) Recycled annual iridium amounts for fixed lifetime $\tau = 10$ and target recycling rates of 97% (baseline scenario),90%,80%, and 70%.

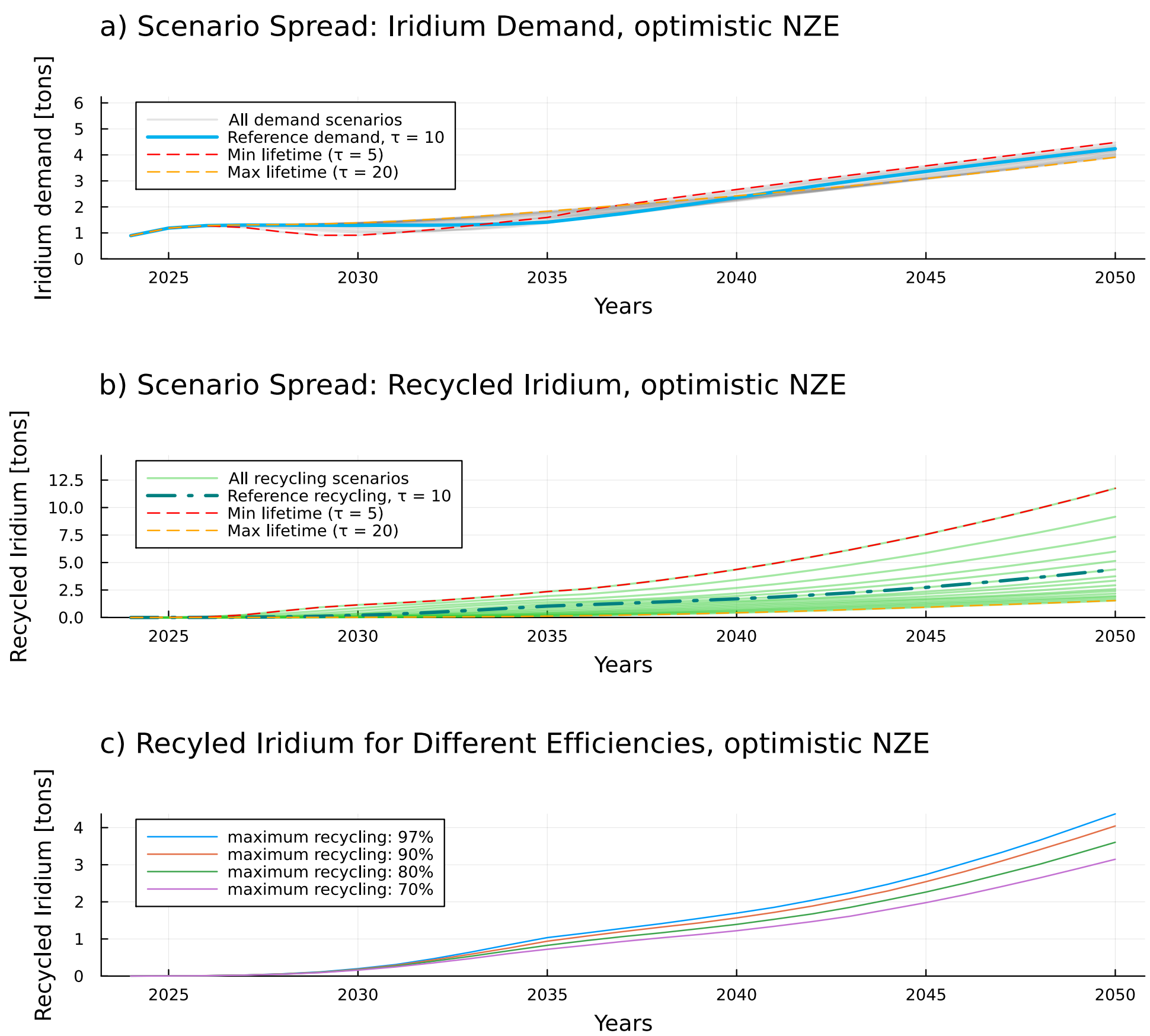

Figure S12: **Optimistic NZE scenario spread:** a) Iridium demand range for varying lifetimes $\tau = 5 - 20$. b) Iridium recycling for varying lifetimes $\tau = 5 - 20$ calculated based on equation 4. c) Recycled annual iridium amounts for fixed lifetime $\tau = 10$ and target recycling rates of 97% (baseline scenario),90%,80%, and 70%.

Bibliography

Brown, S., Cole, L., Cowley, A., Fujita, M., Girardot, N., Grant, S., Jiang, J., Raithatha, R., Ryan, M., Tang, B., 2023. PGM market report. https://matthey.com/documents/161599/404086/PGM+Market+Report+May23.pdf/2f048a72-74a8-8b23-f18e-c875000ed76b?t=1684144507321.

Clapp, M., Zalitis, C.M., Ryan, M., 2023. Perspectives on current and future iridium demand and iridium oxide catalysts for pem water electrolysis. Catalysis Today 420, 114140. doi:https://doi.org/10.1016/j.cattod.2023.114140.

Cueva, S., 2023. Time series analysis in julia. URL: https://datasciencejuliahackers.com/time-series.html.

Eikeng, E., Makhsoos, A., Pollet, B.G., 2024. Critical and strategic raw materials for electrolysers, fuel cells, metal hydrides and hydrogen separation technologies. International Journal of Hydrogen Energy 71, 433–464. URL: https://www.sciencedirect.com/science/article/pii/S036031992401783X, doi:https://doi.org/10.1016/j.ijhydene.2024.05.096.

Holt, C.C., 1957. Forecasting seasonals and trends by exponentially weighted moving averages. Office of Naval Research Research Memorandum 52.

Hyndman, R.J., Athanasopoulos, G., 2018. Forecasting: principles and practice. OTexts.

IEA, PAris, . Hydrogen production and infrastructure projects database. https://www.iea.org/data-and-statistics/data-product/hydrogen-production-and-infrastructure-projects-database. Accessed: 2024-01-16.

IEA, Paris, 2023. Net zero emissions scenario. https://www.iea.org/reports/global-energy-and-climate-model. Accessed: 2024-04-16.

Survey, U.S.G., . Platinum-Group-Metals Survey. https://www.usgs.gov/centers/national-minerals-information-center/platinum-group-metals-statistics-and-information. Accessed: 2024-01-16.

Yu, H., Bonville, L., Jankovic, J., Maric, R., 2020. Microscopic insights on the degradation of a pem water electrolyzer with ultra-low catalyst loading. Applied Catalysis B: Environmental 260, 118194. URL: https://www.sciencedirect.com/science/article/pii/S0926337319309415, doi:https://doi.org/10.1016/j.apcatb.2019.118194.
